# Supplementary figures and images for: Adult medial habenula neurons require GDNF receptor GFRα1 for synaptic stability and function
Source: PLoS Biol. 2021 Nov 8;19(11):e3001350. doi: 10.1371/journal.pbio.3001350 (PMC8601618; doi:10.1371/journal.pbio.3001350)

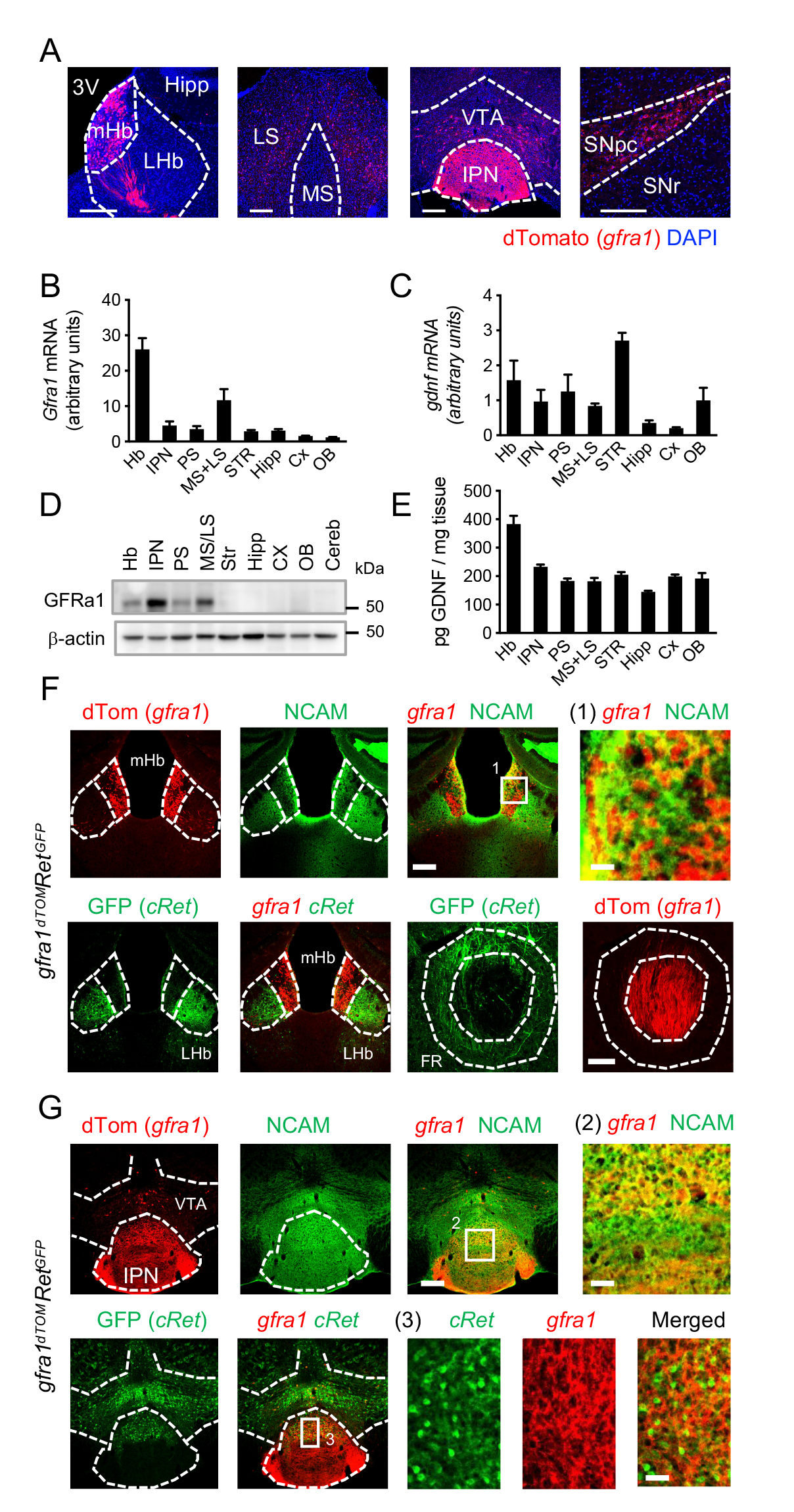

Supplement: S1 Fig — (A) dTomato epifluorescence (red) in coronal sections of Gfra1dTOM mouse brain injected with tamoxifen at 3 months counterstained with DAPI (blue). Scale bars, 200μm. (B, C) Expression levels of gfra1 (B) and gdnf (D) mRNAs quantified by real-time RT-PCR and normalized against 18S in brain areas dissected from 3-month-old C57BL6/J mice (n = 5). (D) Immunoblot of whole protein extracts of 3-month-old C57BL6/J mice probed for GFRα1. β-actin was probed as loading control. (E) Concentration of GDNF in whole protein extracts of 3-month-old C57BL6/J mice quantified by ELISA (N = 3 mice). (F, G) dTomato epifluorescence (red), GFP (green), and NCAM (green) immunolabeling in coronal sections of the mHb and FR (F) and IPN (G) of gfra1dTOMRetGFP mouse injected with tamoxifen at 3 months. Scale bars, 200 μm (mHb) and 30 μm (inset 1), 50 μm (FR), 200 μm (IPN), and 40 μm (insets 2 and 3). The data underlying this figure can be found at https://figshare.com/projects/Raw_Data_Fernandez-Suarez_et_al_2021/123406. Cx, cortex; FR, fasciculus retroflexus; GDNF, glial cell line–derived neurotrophic factor; GFRα1, glial cell–derived neurotrophic factor receptor alpha 1; Hb, habenula; Hipp, hippocampus; IPN, interpeduncular nucleus; LHb, lateral habenula; LS, lateral septum; mHb, medial habenula; MS, medial septum; NCAM, neural cell adhesion molecule; OB, olfactory bulb; PS, posterior septum; SNpc, substantia nigra pars compacta; SNr, substantia nigra reticulata; STR, striatum. (TIF) [file pbio.3001350.s001.tif]

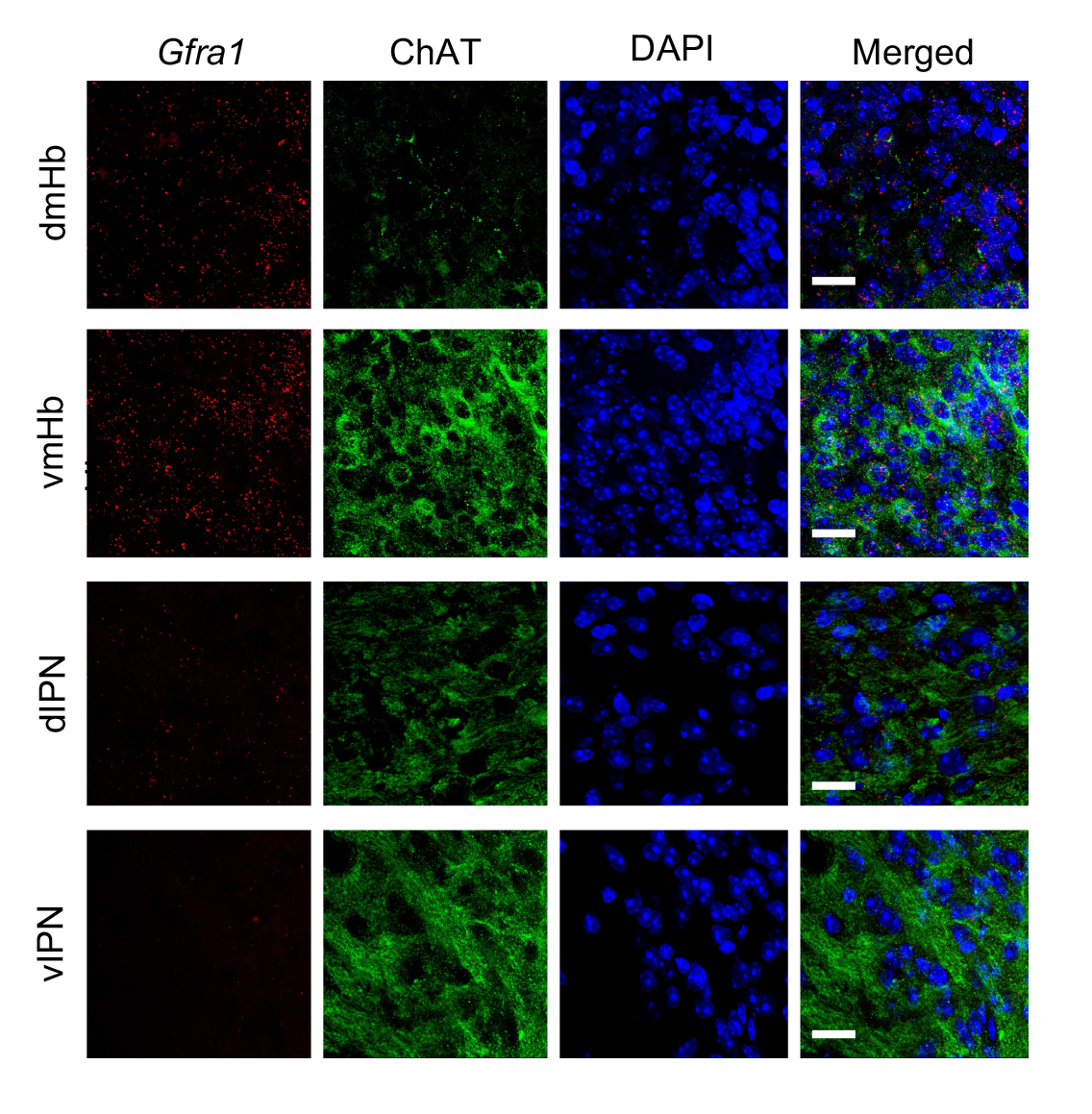

Supplement: S2 Fig — Representative images of the dorsal and vmHb and IPN showing RNAscope for Gfra1 (red), immunolabeling for ChAT (green), and counterstaining with DAPI in brain sections from a C57BL6/J adult mouse. Scale bars, 20 μm. ChAT, choline acetyltransferase; IPN, interpeduncular nucleus; mHb, medial habenula; vmHb, ventral medial habenula. (TIF) [file pbio.3001350.s002.tif]

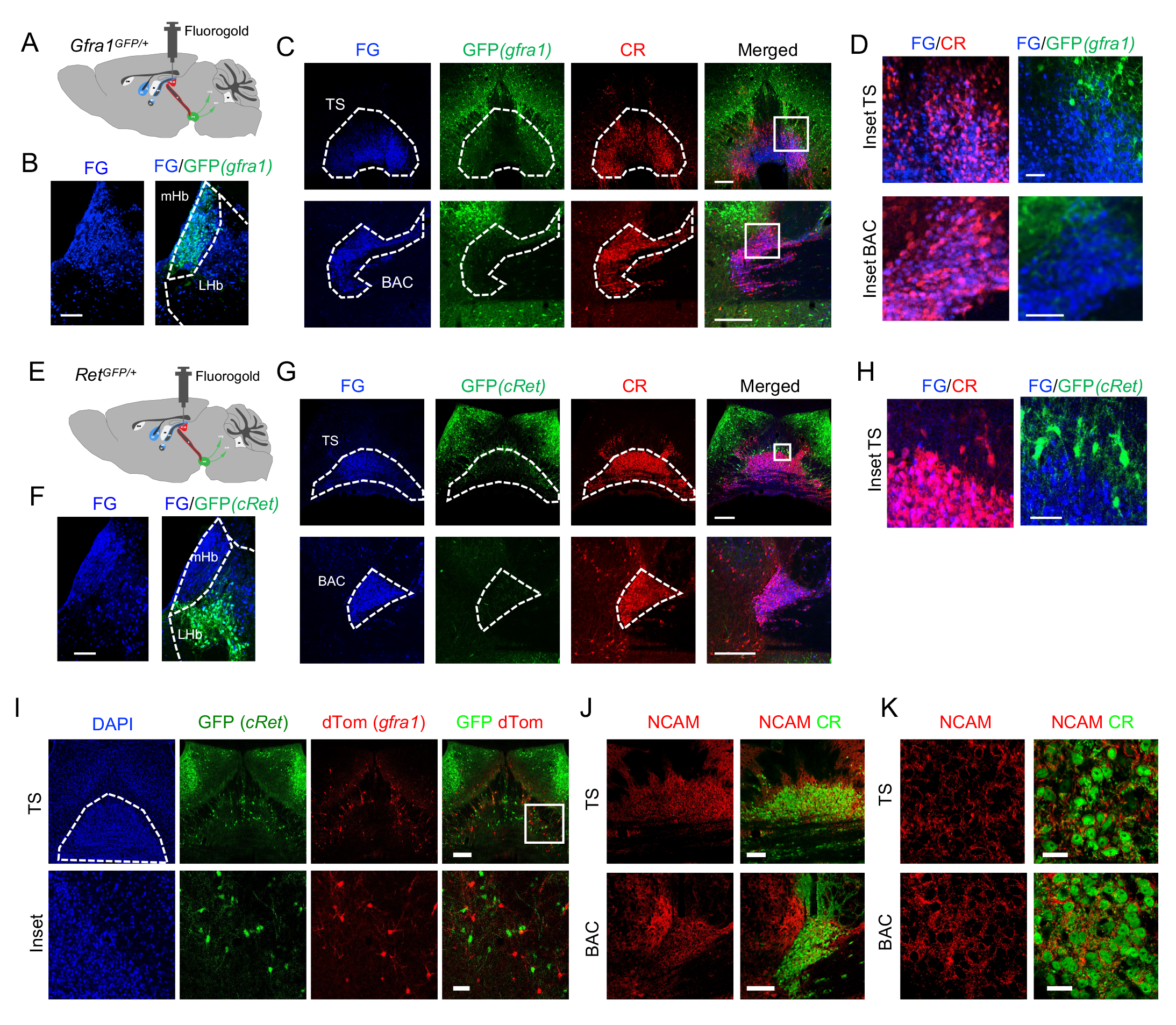

Supplement: S3 Fig — (A, E) Schematic representation of a sagittal brain section of a 3-month-old Gfra1GFP (A) or RetGFP (E) mouse injected with FG in the mHb. (B, F) GFP (green) immunolabeling in coronal sections of the mHb of a Gfra1GFP (B) or a RetGFP (F) mouse injected with FG (blue) in the mHb. Scale bars, 100 μm. (C, D, G, H) CR (red) and GFP (green) immunolabeling in coronal sections of the TS and BAC from a Gfra1GFP (C, D) or a RetGFP (G, H) mouse injected with FG (blue) in the mHb. Scale bars, 200 μm (C, G) and 50 μm (D, H). (I) dTomato epifluorescence (red) and GFP (green) immunolabeling in a coronal section counterstained with DAPI (blue) of the TS of a Gfra1dTOMRetGFP mouse treated with tamoxifen at 3 months. Scale bars, 200 μm and 50 μm (inset). (J, K) NCAM (red) and CR (green) immunolabeling in coronal sections of the TS and BAC of a 3-month-old C57BL6/J mouse. Scale bars, 100 μm (J) and 20 μm (K). BAC, bed nucleus of the anterior commissure; CR, calretinin; FG, fluorogold; GFRα1, glial cell–derived neurotrophic factor receptor alpha 1; mHb, medial habenula; NCAM, neural cell adhesion molecule; TS, triangular septum. (TIF) [file pbio.3001350.s003.tif]

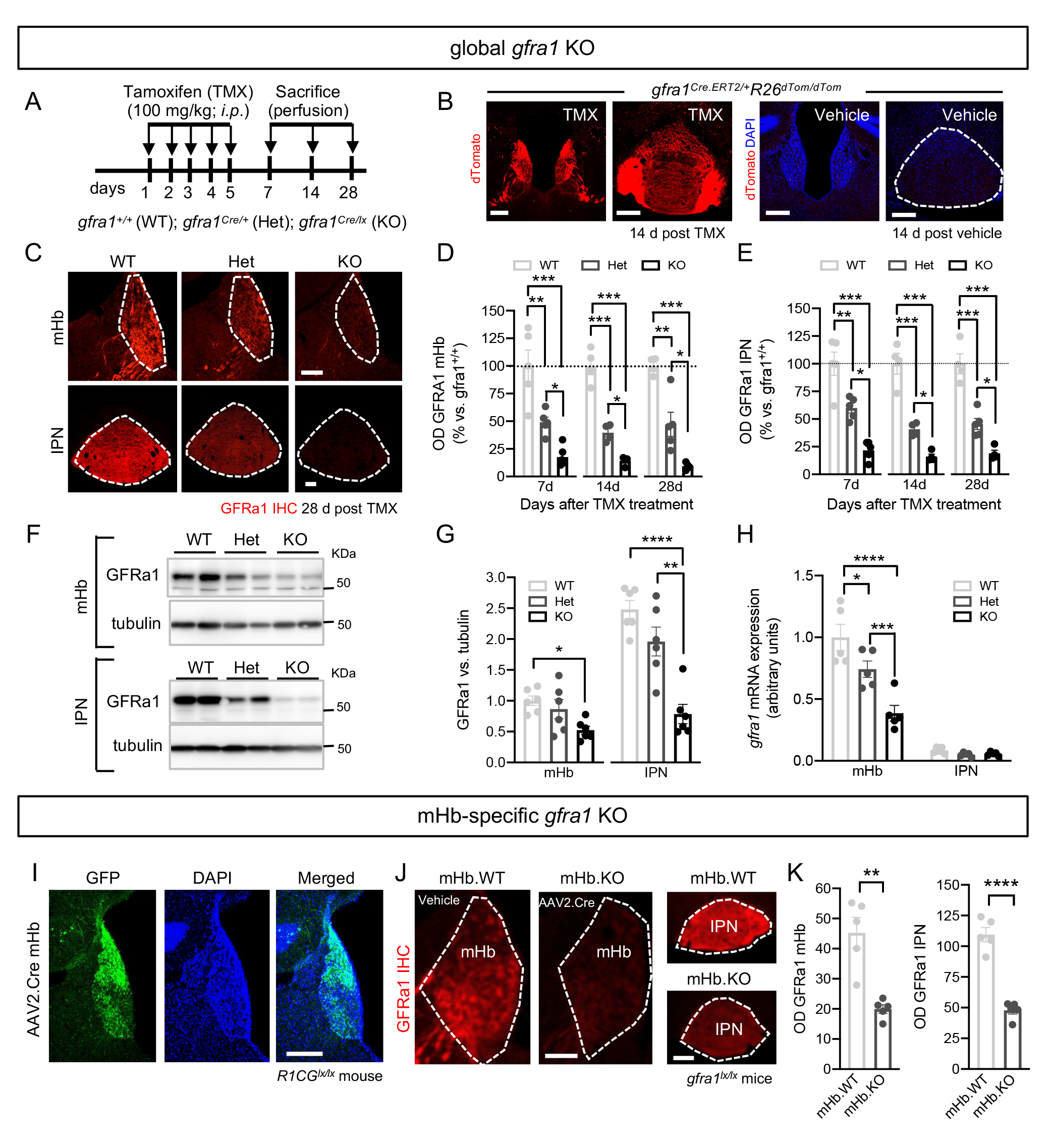

Supplement: S4 Fig — (A) Schematics of the global gfra1 KO mouse model. (B) dTomato epifluorescence (red) in coronal sections of the mHb and the IPN of gfra1dTOM mouse treated with tamoxifen or vehicle at 3 months. Images corresponding to the animal injected with vehicle show also counterstaining with DAPI (blue). Scale bars, 200 μm. (C) Immunolabeling for GFRα1 (red) in coronal sections of the mHb and the IPN of WT, Het, and KO mice. Scale bars, 100 μm. (D, E) Quantification (± SEM) of GFRα1 OD in the mHb (D) and the IPN (E) of WT, Het, and KO mice normalized against the WT group. N = 4–6 animals/group; 1-way ANOVA analysis followed by Tukey post hoc test at each time point; *P < 0.05; **P < 0.01; ***P < 0.001. (F, G) Immunoblots of total protein fraction from mHb and IPN of WT, Het, and KO mice probed for GFRα1 (F) and αtubulin. Quantifications (± SEM) of GFRα1 levels were corrected for αtubulin levels and normalized to levels in WT samples. N = 6 mice per group; 1-way ANOVA followed by Tukey post hoc test; * p < 0.05; **p < 0.01; ****p < 0.0001. (H) Quantification by qPCR of Gfra1 mRNA expression levels (± SEM) in the mHb and the IPN of WT, Het, and KO mice. Gfra1 levels were corrected for 18S levels and normalized to levels in the mHb of WT samples. N = 5 mice per group, 2-way ANOVA followed by Tukey post hoc test; * P < 0.05; ***P < 0.001; ****P < 0.0001. (I) GFP (green) immunolabeling in coronal sections counterstained with DAPI (blue) of the mHb of a R1CGlx/lx mouse injected with an AAV.CMV.Cre virus in the mHb. Scale bar, 100 μm. (J) GFRα1 immunolabeling (red) in coronal sections of the mHb and the IPN of gfra1lx/lx mice injected with vehicle (mHb.WT) or AAV.Cre (mHb.KO) in the mHb. Scale bars, 50 μm (mHb) and 200 μm (IPN). (K) Quantification (± SEM) of GFRα1 OD in the mHb and the IPN of mHb.WT and mHb.KO mice. N = 5 animals per group; Student t test; **P < 0.01; ****P < 0.0001. The data underlying this figure can be found at https://figshare.com/projects/Raw_Data_Fernandez-Suar [file pbio.3001350.s004.tif]

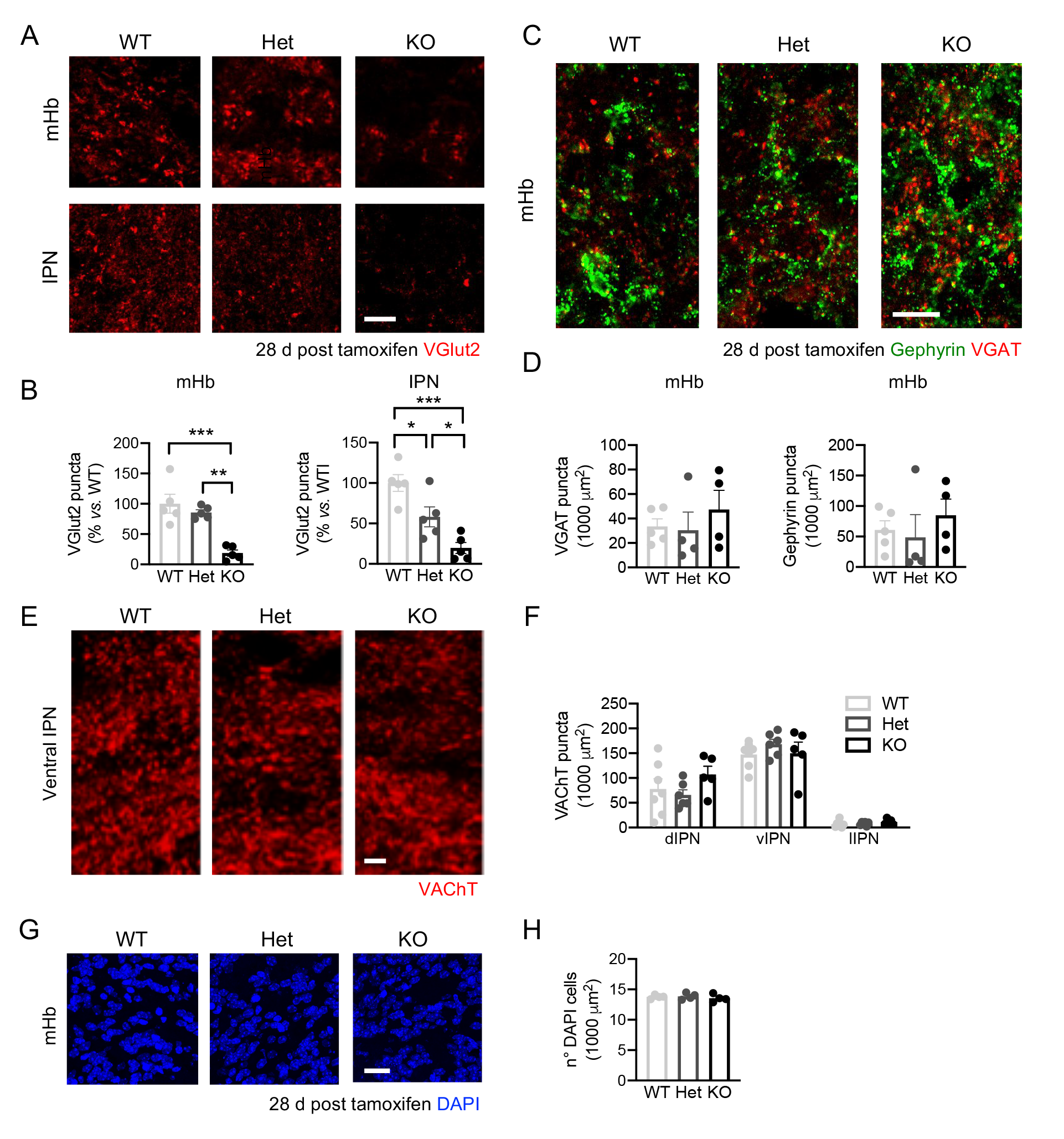

Supplement: S5 Fig — (A) VGlut2 (red) immunostaining in coronal sections of the mHb and the IPN of WT, Het, and KO mice. Scale bar, 10 μm. (B) Quantification (± SEM) of puncta with immunoreactivity for VGlut2 in mHb and IPN. N = 5 mice per group (25–30 images per mouse); 1-way ANOVA followed by Tukey post hoc test; * P < 0.05, ** P < 0.01, *** P < 0.001. (C) VGAT (red) and gephyrin (green) immunostaining in coronal sections of the mHb of WT, Het, and KO mice. Scale bar, 10 μm. (D) Quantification (± SEM) of puncta with immunoreactivity for VGAT and gephyrin in the mHb. N = 4–5 mice per group (25–30 images per mouse). (E) VAChT (red) immunostaining in coronal sections of the IPN of WT, Het, and KO mice. Scale bar, 20 μm. (F) Quantification (± SEM) of puncta with immunoreactivity for VAChT in the dorsal, ventral and lateral IPN. N = 7, 6, and 5 mice in WT, Het, and KO mice, respectively (25–30 images per mouse per structure). (G) DAPI (blue) counterstaining in coronal sections of the mHb of WT, Het, and KO mice. Scale bar, 20 μm. (H) Quantification (± SEM) of number of DAPI cells in the mHb. N = 4 mice per group (32 images per mouse). The data underlying this figure can be found at https://figshare.com/projects/Raw_Data_Fernandez-Suarez_et_al_2021/123406. GFRα1, glial cell–derived neurotrophic factor receptor alpha 1; HET, heterozygous; IPN, interpeduncular nucleus; KO, knockout; mHb, medial habenula; VGlut2, vesicular glutamate transporter 2; WT, wild-type. (TIF) [file pbio.3001350.s005.tif]

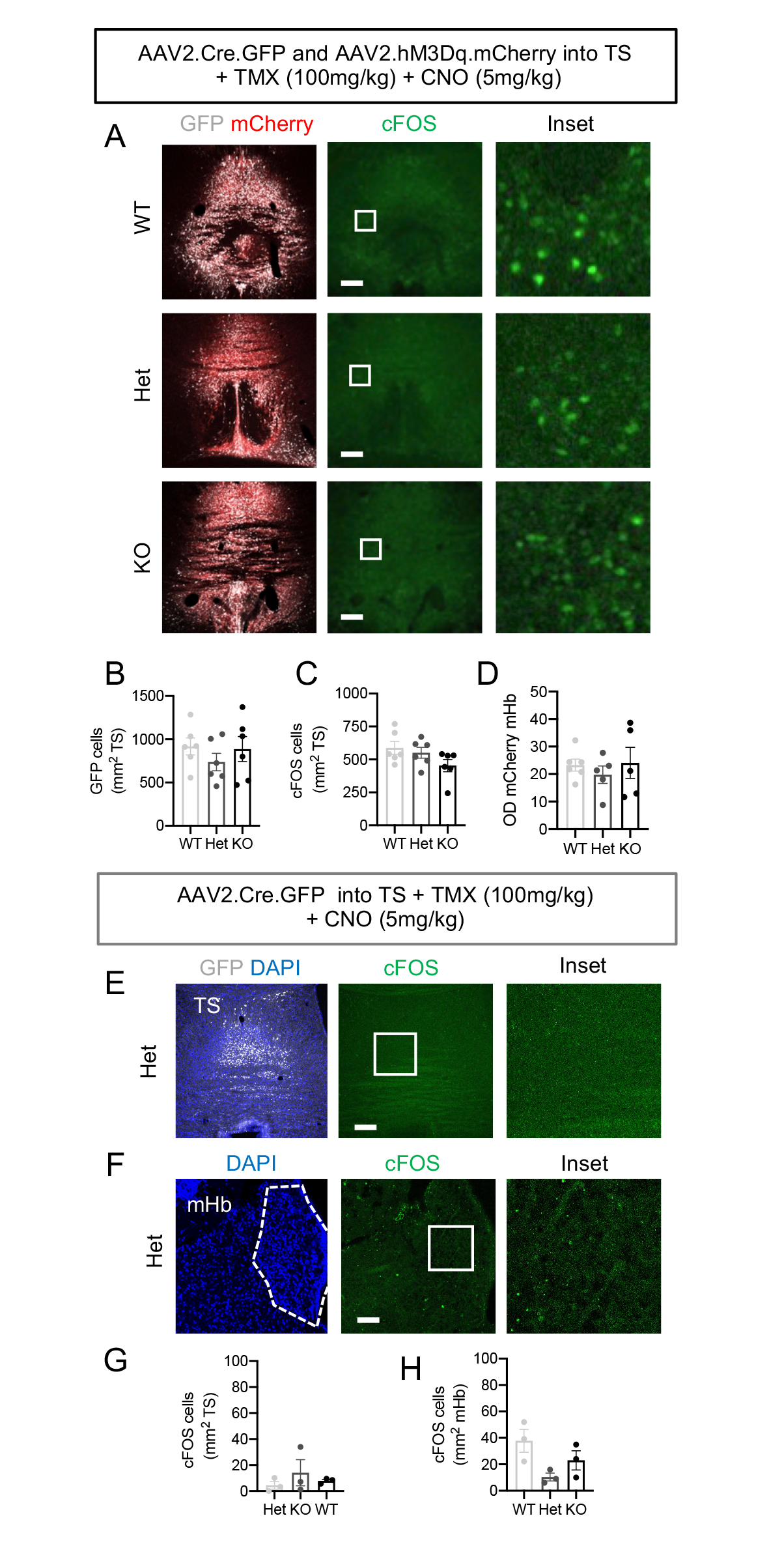

Supplement: S6 Fig — Mice were injected with viral vectors in the TS and treated with tamoxifen and CNO as indicated in each panel. (A–D) Viral transfection efficiency in the TS of WT, Het, and KO mice. mCherry epifluorescence (red), GFP (gray), and immunolabeling for cFOS (green) in coronal sections containing the TS from WT, Het, and KO mice injected with a combination of the Cre and DREADD viruses into the TS (A; scale bars, 200 μm). Graphs show the quantification (± SEM) of the number of GFP+ cells (B) and cFOS+ cells (C) in the TS and the OD for mCherry in the terminals in the mHb (D) of WT, Het, and KO mice. N = 6 mice per group (4–6 TS sections and 6–8 mHb sections per mouse). (E–H) cFos expression after CNO treatment in the absence of the DREADD. GFP (gray), immunolabeling for cFOS (green) and counterstaining with DAPI (blue) in coronal sections containing the TS (E) or the mHb (F) from a Het mouse injected with a AAV.Cre.GFP virus into the TS (scale bar TS = 200 μm; mHb = 100 μm). Graphs show the quantification (± SEM) of the number of cFOS+ cells in the TS (G) and in the mHb (H) after CNO treatment (5 mg/kg; i.p.). N = 3 mice per group (4–6 TS sections TS and 6–8 mHb sections per mouse). The data underlying this figure can be found at https://figshare.com/projects/Raw_Data_Fernandez-Suarez_et_al_2021/123406. CNO, clozapine N-oxide; DREADD, designer receptor exclusively activated by designer drug; HET, heterozygous; KO, knockout; mHb, medial habenula; OD, optical density; TS, triangular septum; WT, wild-type. (TIF) [file pbio.3001350.s006.tif]

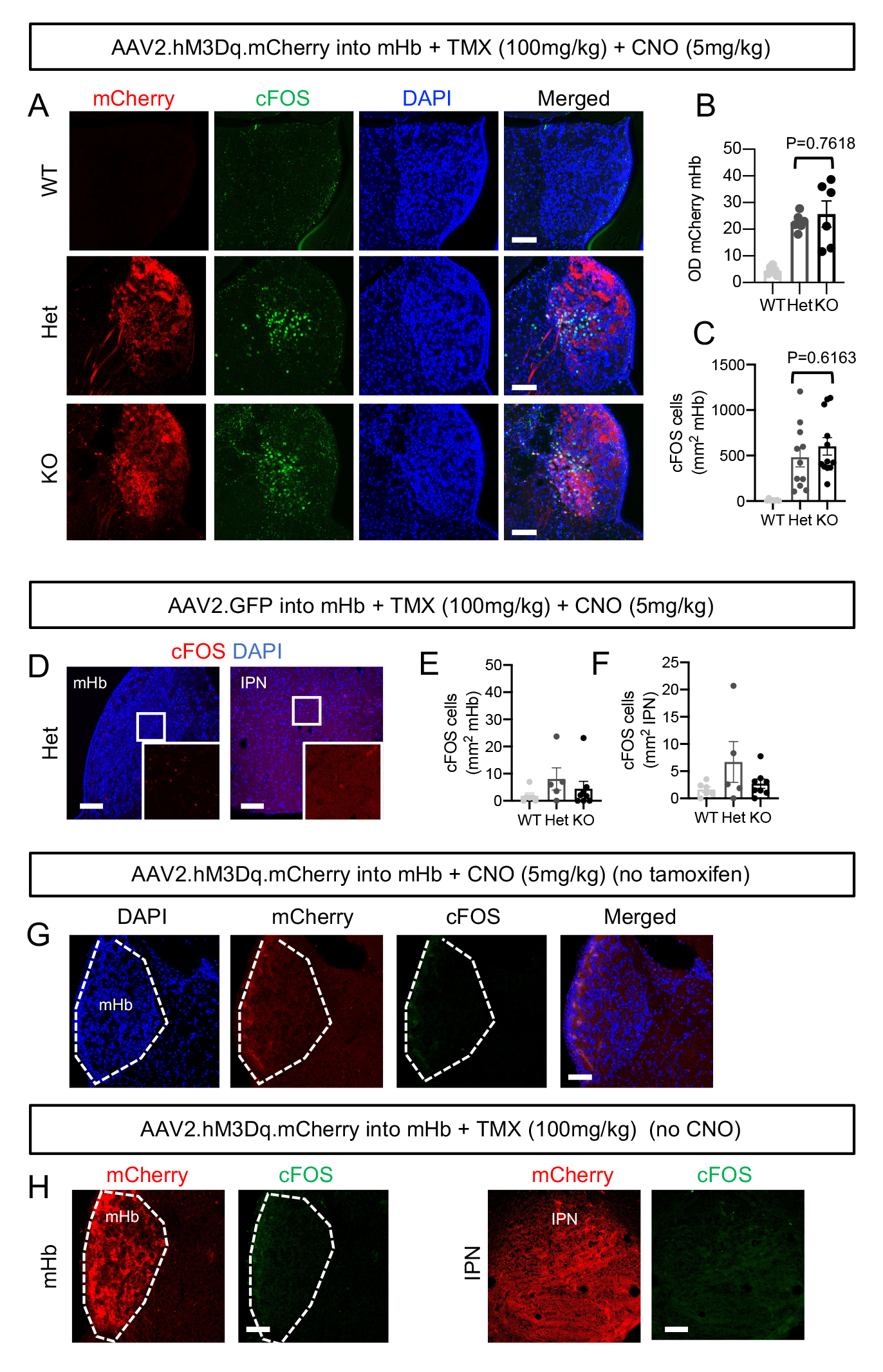

Supplement: S7 Fig — Mice were injected with viral vectors in the mHb and treated with tamoxifen, CNO, or vehicles as indicated in each panel. (A–C) Viral transfection efficiency in the mHb of WT, Het, and KO mice. mCherry epifluorescence (red), immunolabeling for c-Fos (green), and counterstaining with DAPI (blue) in coronal sections containing the mHb from WT, Het, and KO mice injected with the DREADD virus into the mHb (A; scale bars, 100 μm). Graphs show the quantification (± SEM) of the OD for mCherry (B, N = 6 mice per group, 10–12 sections per mouse) and the number of cFOS+ cells (C, N = 7, 11, 12) in the mHb of WT, Het, and KO mice. (D–F) cFOS expression in the mHb and IPN after CNO treatment in the absence of the DREADD. Immunolabeling for cFOS (red) and counterstaining with DAPI (blue) in the mHb and IPN (D) of a mouse injected with an AAV2.GFP virus in the mHb and treated with CNO. Graphs show the quantification (± SEM) of cFOS+ cells in the mHb (E) and the IPN (F). Scale bars, 70 μm (mHb) and 100 μm (IPN). (G) mCherry epifluorescence (red), cFOS immunostaining (green), and counterstaining with DAPI in the mHb of a mouse injected with the DREADD virus and treated with the vehicle of tamoxifen showing no expression of the DREADD neither cFOS after CNO treatment. Scale bar, 100 μm. (H) mCherry epifluorescence (red) and cFos immunostaining (green) in the mHb and the IPN of a mouse injected with the DREADD virus showing no cFOS expression in the absence of CNO. Scale bars, 100 μm (mHb) and 200 μm (TS). The data underlying this figure can be found at https://figshare.com/projects/Raw_Data_Fernandez-Suarez_et_al_2021/123406. CNO, clozapine N-oxide; DREADD, designer receptor exclusively activated by designer drug; HET, heterozygous; IPN, interpeduncular nucleus; KO, knockout; mHb, medial habenula; OD, optical density; TS, triangular septum; WT, wild-type. (TIF) [file pbio.3001350.s007.tif]

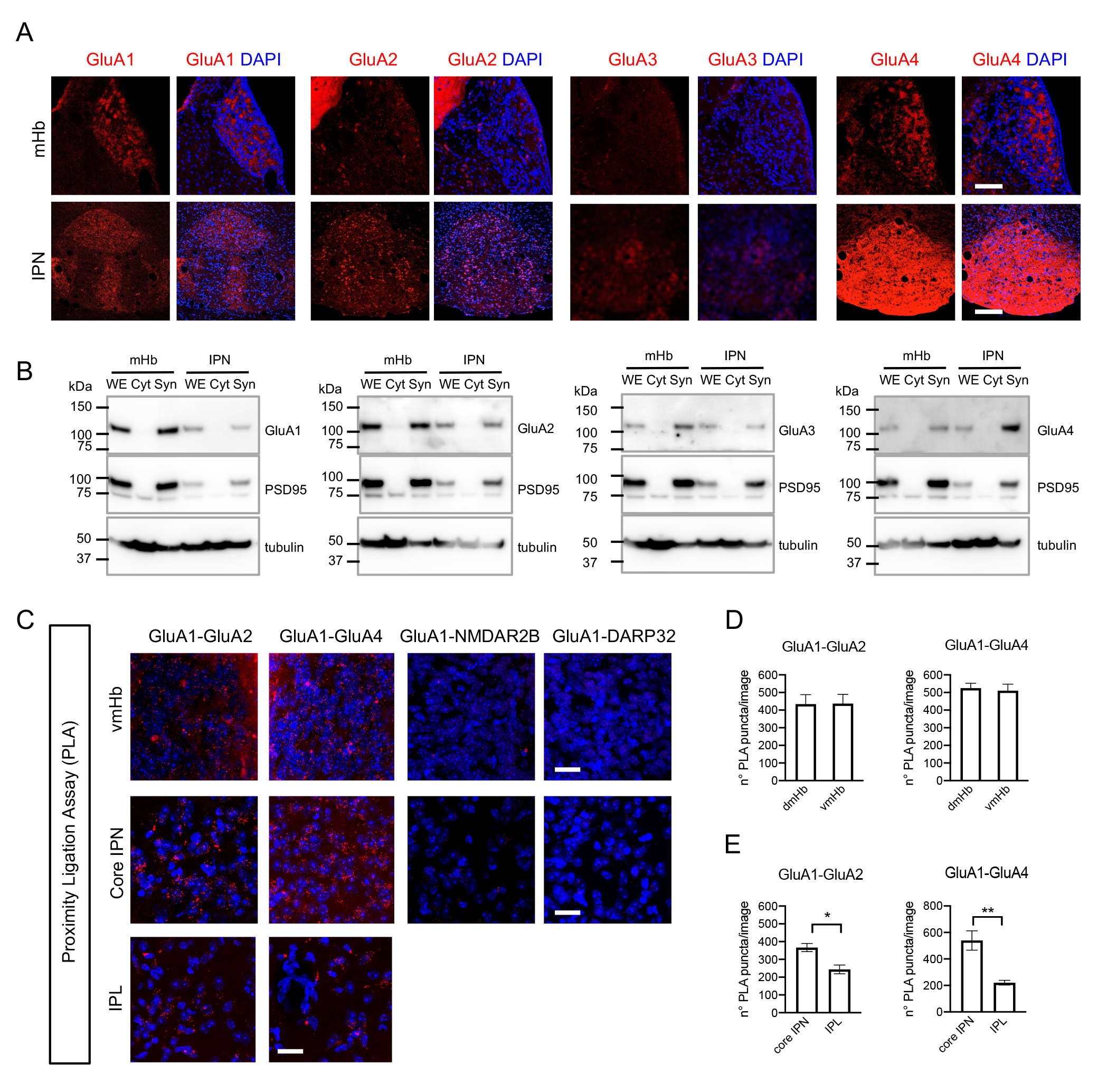

Supplement: S8 Fig — (A) GluA1-4 immunostaining (red) and counterstaining in DAPI (blue) in coronal sections of the mHb and the IPN of a 3-month-old C57BL6/J mouse. Scale bars, 150 μm (mHb) and 300 μm (IPN). (B) Immunoblots of WE, Cyt, and Syn protein extracts from the mHb and IPN of 3-month-old C57BL6/J mice probed against GluA1-4. PSD95 and tubulin were probed as loading controls. (C) PLA signals (red) for GluA1-GluA2 and GluA1-GluA4 complexes in coronal sections of mHb and IPN from 3-month-old C57BL6/J mice. Counterstaining with DAPI appears in blue. NMDAR2B and DARP32 were used as negative controls. Scale bars, 20 μm. (D, E) Quantification (± SEM) of PLA puncta for GluA1-GluA2 and GluA1-GluA4 complexes in the dorsal and vmHb (D) and the lateral and core IPN (E) from 3-month-old C57BL6/J mice. N = 4 mice (16 images per mHb and 18 images per IPN). Student t test; * P < 0.05; ** P < 0.01. The data underlying this figure can be found at https://figshare.com/projects/Raw_Data_Fernandez-Suarez_et_al_2021/123406. Cyt, cytosolic protein fraction; IPN, interpeduncular nucleus; mHb, medial habenula; PLA, proximity ligation assay; Syn, synaptosome protein fraction; vmHb, ventral medial habenula; WE, whole protein extract. (TIF) [file pbio.3001350.s008.tif]

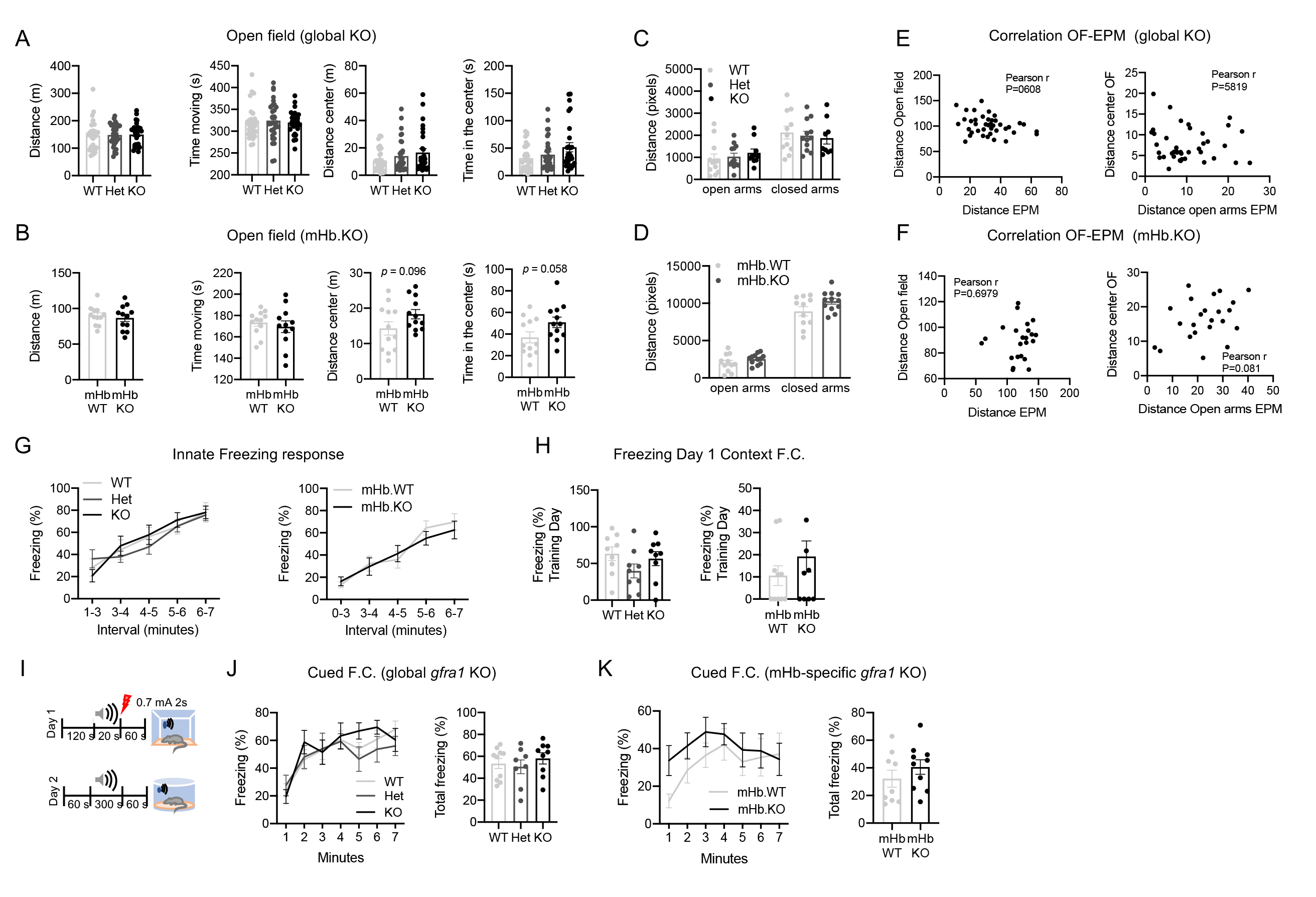

Supplement: S9 Fig — (A, B) Total distance, time moving and distance, and time spent in the center of the open field arena (± SEM) for WT, Het, and KO mice (A) and mHb.WT and mHb.KO mice (B). N = 28–31 mice per group, Kruskal–Wallis test followed by Dunn multiple comparison test (A). N = 12 mice per group, Student t test (B). (C, D) Distance covered in the open and closed arms of the EPM for WT, Het, and KO mice (N = 10–12 animals per group) (C) and mHb.WT and mHb.KO mice (N = 11–12 animals per group) (D). (E, F) Correlation analysis between the distance covered in the open field test (OF) and in the EPM for WT, Het, and KO mice (E) and mHb.WT and mHb.KO mice (F). (G) Freezing responses (± SEM) after 3 consecutive foot shocks for WT, Het, and KO mice (N = 10–13 mice per group) and mHb.WT and mHb.KO mice (N = 12 mice per group). Two-way ANOVA followed by Tukey post hoc test. (H) Percentage of freezing in the training day of the context fear conditioning test for WT, Het, and KO mice (N = 8–10 mice per group, 1-way ANOVA) and mHb.WT and mHb.KO mice (N = 9–10 mice per group, Student t test). (I, J, K) Schematic (I), freezing timecourse and total freezing time (± SEM) for WT, Het, and KO mice (J) and mHb.WT and mHb.KO mice (K) in the cued fear conditioning test. N = 8–10 mice per group, 2-way ANOVA and 1-way ANOVA (J). N = 9–10 mice per group, 2-way ANOVA and Student t test (K). The data underlying this figure can be found at https://figshare.com/projects/Raw_Data_Fernandez-Suarez_et_al_2021/123406. EPM, elevated plus maze; HET, heterozygous; KO, knockout; mHb, medial habenula; WT, wild-type. (TIF) [file pbio.3001350.s009.tif]
