# Supplementary material for: Adult medial habenula neurons require GDNF receptor GFRα1 for synaptic stability and function
Source: PLoS Biol. 2021 Nov 8;19(11):e3001350. doi: 10.1371/journal.pbio.3001350 (PMC8601618; doi:10.1371/journal.pbio.3001350)
Supplement: S1 Table — (PDF) [file pbio.3001350.s010.pdf]

S1 Table. Statistics Figures 1-6

Figure	Panel	Graph	N (*)	Statistical Test	F value	P value	Group Comparitions		
							p WT vs Het	*p* WT vs KO	*p* Het vs KO
2	B	mHb	4, 4, 4	1-way ANOVA, Tukey's	6.3160	0.0193	0.2774	0.0154	0.1924
2	B	IPN	6, 6, 6	1-way ANOVA, Tukey's	4.5610	0.0336	0.5749	0.0291	0.1683
2	C	mHb	5, 5, 5	1-way ANOVA, Tukey's	5.7670	0.0176	0.9745	0.0377	0.0256
2	C	IPN	5, 5, 5	1-way ANOVA, Tukey's	13.190	0.0009	0.0104	0.0008	0.3500
2	F	synapsin I	7, 7	Unpaired t test		0.0239			
2	F	PSD93	6, 6	Unpaired t test		0.0321			
2	F	Vglut1	6, 6	Unpaired t test		<0.0001			
2	F	Vglut2	6, 6	Unpaired t test		0.0144			
2	H	synapsin I	7, 7	Unpaired t test		0.0442			
2	H	PSD93	5, 5	Unpaired t test		0.0507			
2	H	Vglut1	5, 5	Unpaired t test		0.0242			
2	H	Vglut2	5, 5	Unpaired t test		0.2919			
3	B	N° synapses	3, 3, 3	1-way ANOVA, Tukey's	12.870	0.0068	0.8428	0.0084	0.0153
3	B	N° vesicles	3, 3, 3	1-way ANOVA, Tukey's	7.7980	0.0214	0.0537	0.0230	0.7674
3	B	PSD lenght	3, 3, 3	1-way ANOVA, Tukey's	1.3700	0.3235	0.3682	0.4011	0.9969
3	B	Synaptic cleft	3, 3, 3	1-way ANOVA, Tukey's	3.4020	0.1029	0.1248	0.1564	0.9829
3	D	N° synapses	3, 3, 3	1-way ANOVA, Tukey's	8.1680	0.0194	0.9806	0.0267	0.0334
3	D	N° vesicles	3, 3, 3	1-way ANOVA, Tukey's	39.370	0.0004	0.0026	0.0003	0.0681
3	D	PSD lenght	3, 3, 3	1-way ANOVA, Tukey's	0.0639	0.9388	0.9766	0.9334	0.9880
3	D	Synaptic cleft	3, 3, 3	1-way ANOVA, Tukey's	1.7680	0.2490	0.2251	0.5828	0.6940
4	C	TS-mHb	6, 6, 6	1-way ANOVA, Tukey's	6.6650	0.0085	0.0146	0.0193	0.9892
4	E	mHb-IPN	7, 12, 11	1-way ANOVA, Tukey's	8.7530	0.0012	0.0014	0.3408	0.0210
4	G	EPSC -60mV	(#) 10, 11, 14	Kruskal-Wallis, Dunn's		0.0446	0.3625	0.0389	>0.9999
4	H	EPSC +50mV	(#) 10, 11, 14	1-way ANOVA, Tukey's	0.9099	0.4130	0.7406	0.8515	0.3805
4	I	RI AMPA	(#) 10, 11, 14	Kruskal-Wallis, Dunn's		0.0005	0.2706	0.0003	0.1061
4	J	EPSC NMDA	(#) 9, 17, 18	1-way ANOVA, Tukey's	0.3690	0.6938	0.7395	0.6964	0.9967
5	A	GluA1	9, 9, 9	1-way ANOVA, Tukey's	3.3150	0.0535	0.9318	0.1269	0.0628
5	A	GluA2	10,10,10	1-way ANOVA, Tukey's	3.9460	0.0314	0.2645	0.0245	0.4612
5	A	GluA3	10,10,10	1-way ANOVA, Tukey's	0.1604	0.8526	0.8415	0.9365	0.9746
5	A	GluA4	10,10,10	1-way ANOVA, Tukey's	0.1793	0.8368	0.8585	0.9998	0.8676
5	B	GluA1	10,10,10	1-way ANOVA, Tukey's	6.1170	0.0064	0.0662	0.0055	0.5391
5	B	GluA2	10,10,10	1-way ANOVA, Tukey's	0.2696	0.7657	0.8976	0.7489	0.9563
5	B	GluA3	10, 9, 10	1-way ANOVA, Tukey's	0.3238	0.7263	0.9322	0.7041	0.9056
5	B	GluA4	10, 10, 9	1-way ANOVA, Tukey's	6.6390	0.0047	0.0917	0.0035	0.3176
5	D	GluA1 Ser831	13,13	Unpaired t test		0.3274			
5	D	GluA1 Ser845	13,13	Unpaired t test		0.0166			
5	D	GluA1 total	13,13	Unpaired t test		0.7475			
5	F	GluA1 Ser831	15,15	Unpaired t test		0.0785			
5	F	GluA1 Ser845	15,15	Unpaired t test		0.5203			
5	F	GluA1 total	15,15	Unpaired t test		0.0029			
5	H	GluA1-GluA2 mHb	5, 5	Unpaired t test		0.0116			
5	J	GluA1-GluA4 mHb	5, 5	Unpaired t test		0.9262			
6	A	Total entries	11,12,10	1-way ANOVA, Tukey's	0.9369	0.4030	0.8194	0.7273	0.3699
6	A	Time open arms	11,12,10	1-way ANOVA, Tukey's	5.8970	0.0069	0.9113	0.0262	0.0086
6	A	Entries open arms	11,12,10	1-way ANOVA, Tukey's	3.5690	0.0407	0.8302	0.0389	0.1373
6	B	Total entries	11, 12	Unpaired t test		0.1677			
6	B	Time open arms	11, 12	Unpaired t test		0.0463			
6	B	Entries open arms	11, 12	Unpaired t test		0.1665			
6	G	Total freezing	10, 8, 9	1-way ANOVA, Tukey's	0.2943	0.7477	0.8516	0.9703	0.7365
6	H	Total freezing	9, 10	Unpaired t test		0.0310			

* N values are always pesented as (i) WT, Het ,KO or (ii) mHb.WT, mHb.KO mice as appropiate for each graph

N values represent WT, Het and KO cells
