# Supplementary material for: Adult medial habenula neurons require GDNF receptor GFRα1 for synaptic stability and function
Source: PLoS Biol. 2021 Nov 8;19(11):e3001350. doi: 10.1371/journal.pbio.3001350 (PMC8601618; doi:10.1371/journal.pbio.3001350)
Supplement: S2 Table — (PDF) [file pbio.3001350.s011.pdf]

S2 Table. 2-Way ANOVA Analysis Figures 1-6

								Group Comparitions			
**Figure**	**Panel**	**Graph**	**N (*)**	**Statistical Test**	**Source of Variat**	**F value**	**P value**	**Variable**	***p* WT vs Het**	***p* WT vs KO**	***p* Het vs KO**
5	H	GluA1-GluA2 IPN	5, 5	2-way ANOVA, Bonferroni's	Genotype	F (1, 16) = 1.04	0.3230	core IPN		0.0197	
		Global KO			Subnuclei	F (1, 16) = 155.	< 0.0001	lateral IPN		0.3130	
		WT *vs.* KO			Interaction	F (1, 16) = 9.75	0.0066				
5	J	GluA1-GluA4 IPN	5, 5	2-way ANOVA, Bonferroni's	Genotype	F (1, 16) = 4.27	0.0306	core IPN		0.0127	
		Global KO			Subnuclei	F (1, 16) = 62.3	< 0.0001	lateral IPN		> 0.9999	
		WT *vs.* KO			Interaction	F (1, 16) = 5.62	0.0552				
6	D	Passive avoidance	12, 11, 8	2-way ANOVA, Bonferroni's	Genotype	F (2, 56) = 4.31	0.0180	Day 1	> 0.9999	> 0.9999	> 0.9999
		Global KO			Day	F (1, 56) = 45.0	< 0.0001	Day 2	0.1824	0.0024	0.2499
					Interaction	F (2, 56) = 2.22	0.1180				
6	E	Passive avoidance	10, 11	2-way ANOVA, Bonferroni's	Animal group	F (1, 38) = 4.71	0.0362	Day 1		0.9515	
		mHb.KO			Day	F (1, 38) = 31.4	< 0.0001	Day 2		0.0163	
					Interaction	F (1, 38) = 3.14	0.0410				
6	G	Context FC	10, 8, 9	2-way ANOVA, Bonferroni's	Genotype	F (2, 72) = 0.55	0.5774	Min 1	> 0.9999	> 0.9999	> 0.9999
		Freezing per min			Minute	F (2, 72) = 11.1	< 0.0001	Min 2	> 0.9999	> 0.9999	> 0.9999
		Global KO			Interaction	F (4, 72) = 0.12	0.9718	Min 3	> 0.9999	> 0.9999	> 0.9999
6	H	Context FC	9, 10	2-way ANOVA, Bonferroni's	Animal group	F (1, 51) = 11.3	0.0015	Min 1		0.5942	
		Freezing per min			Minute	F (2, 51) = 4.21	0.0203	Min 2		0.1921	
		mHb.KO			Interaction	F (2, 51) = 0.72	0.4912	Min 3		0.0197	

* N values are always pesented as (i) WT, Het ,KO or (ii) mHb.WT, mHb.KO as appropiate for each graph
