# Supplementary material for: Adult medial habenula neurons require GDNF receptor GFRα1 for synaptic stability and function
Source: PLoS Biol. 2021 Nov 8;19(11):e3001350. doi: 10.1371/journal.pbio.3001350 (PMC8601618; doi:10.1371/journal.pbio.3001350)
Supplement: S3 Table — (PDF) [file pbio.3001350.s012.pdf]

S3 Table. Statistics Figures S1-S9

Figure	Panel	Graph	N (*)	Statistical Test	F value	P value	Group Comparitions		
							p WT vs Het	*p* WT vs KO	*p* Het vs KO
S4	D	7d	5, 5, 5	1-way ANOVA, Tukey's	23.33	<0.0001	0.0043	<0.0001	0.0438
S4	D	14d	4, 4, 4	1-way ANOVA, Tukey's	70.92	<0.0001	<0.0001	<0.0001	0.0184
S4	D	28d	4, 5, 4	1-way ANOVA, Tukey's	31.61	<0.0001	0.0017	<0.0001	0.0150
S4	E	7d	5, 5, 6	1-way ANOVA, Tukey's	26.40	<0.0001	0.0058	<0.0001	0.0149
S4	E	14d	4, 4, 4	1-way ANOVA, Tukey's	54.32	<0.0001	0.0001	<0.0001	0.0375
S4	E	28d	4, 5, 4	1-way ANOVA, Tukey's	43.78	<0.0001	0.0002	<0.0001	0.0252
S4	G	mHb	6, 6, 6	1-way ANOVA, Tukey's	5.268	0.0185	0.6522	0.0171	0.0939
S4	G	IPN	6, 6, 6	1-way ANOVA, Tukey's	22.59	<0.0001	0.1425	<0.0001	0.0011
S4	K	mHb	5, 5	Unpaired t test		0.0014			
S4	K	IPN	5, 5	Unpaired t test		<0.0001			
S5	B	mHb	5, 5, 5	1-way ANOVA, Tukey's	19.47	0.0002	0.5704	0.0002	0.0011
S5	B	IPN	5, 5, 5	1-way ANOVA, Tukey's	15.86	0.0004	0.0314	0.0003	0.0472
S5	D	VGAT	5, 4, 4	1-way ANOVA, Tukey's	0.5182	0.6107	0.9831	0.6998	0.6258
S5	D	Gephyrin	5, 4, 4	1-way ANOVA, Tukey's	0.4607	0.6436	0.9442	0.7903	0.6289
S5	H	DAPI cells	4, 4, 4	1-way ANOVA, Tukey's	0.3994	0.6820	0.9706	0.8074	0.6750
S6	B	GFP cells	6, 6, 6	1-way ANOVA, Tukey's	0.6912	0.5163	0.5298	0.9813	0.6407
S6	C	cFos cells	6, 6, 6	1-way ANOVA, Tukey's	2.3290	0.1315	0.8394	0.1262	0.3099
S6	D	OD mCherry	6, 6, 6	1-way ANOVA, Tukey's	0.5494	0.5885	0.8432	0.8781	0.5597
S6	G	cFos TS	3, 3, 3	1-way ANOVA, Tukey's	0.6462	0.5570	0.9169	0.7603	0.5367
S6	H	cFos mHb	3, 3, 3	1-way ANOVA, Tukey's	4.1700	0.0732	0.0270	0.3338	0.4303
S7	B	OD mCherry	6, 6, 6	1-way ANOVA, Tukey's	15.040	0.0003	0.0015	0.0004	0.7618
S7	C	cFos mHb	7, 11, 12	1-way ANOVA, Tukey's	8.8120	0.0011	0.0089	0.0009	0.6163
S7	E	cFos mHb	6, 5, 8	1-way ANOVA, Tukey's	1.0750	0.3648	0.3333	0.7721	0.6474
S7	F	cFos IPN	6, 5, 8	1-way ANOVA, Tukey's	1.8560	0.1885	0.1892	0.8995	0.3014
S8	D	GluA1-GluA2	4, 4	Unpaired t test		0.9721			
S8	D	GluA1-GluA4	4, 4	Unpaired t test		0.7702			
S8	E	GluA1-GluA2	4, 4	Unpaired t test		0.0106			
S8	E	GluA1-GluA4	4, 4	Unpaired t test		0.0054			
S9	A	Distance	30, 31, 28	Kruskal Wallis, Dunn's		0.9363	0.9679	0.9332	0.9923
S9	A	Time moving	30, 31, 28	Kruskal Wallis, Dunn's		0.5634	0.8762	>0.9999	>0.9999
S9	A	Dist center	30, 31, 28	Kruskal Wallis, Dunn's		0.3050	0.9808	0.3865	>0.9999
S9	A	Time center	30, 31, 28	Kruskal Wallis, Dunn's		0.1875	>0.9999	0.2047	0.8197
S9	B	Distance	12, 12	Unpaired t test		0.6230			
S9	B	Time moving	12, 12	Unpaired t test		0.5521			
S9	B	Dist center	12, 12	Unpaired t test		0.0966			
S9	B	Time center	12, 12	Unpaired t test		0.0588			
S9	H	Global KO	10, 8, 9	1-way ANOVA, Tukey's	1.6450	0.2140	0.2040	0.8698	0.4327
S9	H	mHb.KO	9, 10	Unpaired t test		0.3110			
S9	J	Time freezing	10, 8, 9	1-way ANOVA, Tukey's	0.52	0.6039	0.9184	0.7938	0.5843
S9	K	Time freezing	9, 10	Unpaired t test		0.3052			

* N values are always pesented as (i) WT, Het ,KO or (ii) mHb.WT, mHb.KO mice as appropiate for each graph
