# Supplementary material for: Adult medial habenula neurons require GDNF receptor GFRα1 for synaptic stability and function
Source: PLoS Biol. 2021 Nov 8;19(11):e3001350. doi: 10.1371/journal.pbio.3001350 (PMC8601618; doi:10.1371/journal.pbio.3001350)
Supplement: S4 Table — (PDF) [file pbio.3001350.s013.pdf]

S4 Table. 2-Way ANOVA Analysis Figures S1-S9

								Group Comparitions			
**Figure**	**Panel**	**Graph**	**N (*)**	**Statistical Test**	**Source of Vari**	**F value**	**P value**	**Variable**	***p* WT vs Het**	***p* WT vs KO**	***p* Het vs KO**
S4	H	*gfra1* mRNA	5, 5, 5	2-way ANOVA, Bonferroni's	Genotype	F(2,24)=15.52	<0.0001	mHb	0.0126	<0.0001	0.0006
					Subnuclei	F(1,24)=187.6	<0.0001	IPN	>0.9999	>0.9999	>0.9999
					Interaction	F(2,24)=13.17	0.0001				
S5	F	VAChT IPN	7, 6, 5	2-way ANOVA, Bonferroni's	Genotype	F (2, 45) = 0.6903	0.5066	dorsal	>0.9999	0.3426	0.0980
					Subnuclei	F (2, 45) = 98.32	<0.0001	ventral	0.7137	>0.9999	>0.9999
					Interaction	F (4, 45) = 1.341	0.2695	lateral	>0.9999	>0.9999	>0.9999
S9	J	Cued FC	10, 8, 9	2-way ANOVA, Bonferroni's	Genotype	F (2, 168) = 1.292	0.2774	Min 1	>0.9999	>0.9999	>0.9999
		Freezing per min			Minute	F (6, 168) = 10.11	<0.0001	Min 2	>0.9999	0.6188	0.9300
		Global KO			Interaction	F (12, 168) = 0.628	0.6288	Min 3	>0.9999	>0.9999	>0.9999
								Min 4	>0.9999	>0.9999	>0.9999
								Min 5	>0.9999	0.6105	0.1711
								Min 6	>0.9999	>0.9999	0.4010
								Min 7	0.7413	>0.9999	>0.9999
S9	K	Cued FC	9, 10	2-way ANOVA, Bonferroni's	Animal group	F (1, 119) = 3.917	0.0501	Min 1		0.3559	
		Freezing per min			Minute	F (6, 119) = 1.537	0.1719	Min 2		0.8720	
		mHb.KO			Interaction	F (6, 119) = 0.476	0.8248	Min 3		0.8978	
								Min 4		0.9991	
								Min 5		0.9975	
								Min 6		>0.9999	
								Min 7		>0.9999	

* N values are always pesented as (i) WT, Het ,KO or (ii) mHb.WT, mHb.KO mice as appropiate for each graph
