# Supplementary material for: Adult medial habenula neurons require GDNF receptor GFRα1 for synaptic stability and function
Source: PLoS Biol. 2021 Nov 8;19(11):e3001350. doi: 10.1371/journal.pbio.3001350 (PMC8601618; doi:10.1371/journal.pbio.3001350)

WESTERN-BLOTS MAIN FIGURES

Figure 1H

Samples: mHb and IPN dissected from C57BL6/J mice. The whole extract (WE), the cytosolic (Cyt) and the synaptosome (Syn) fraction were extracted and 20 micrograms of samples loaded in each lane.

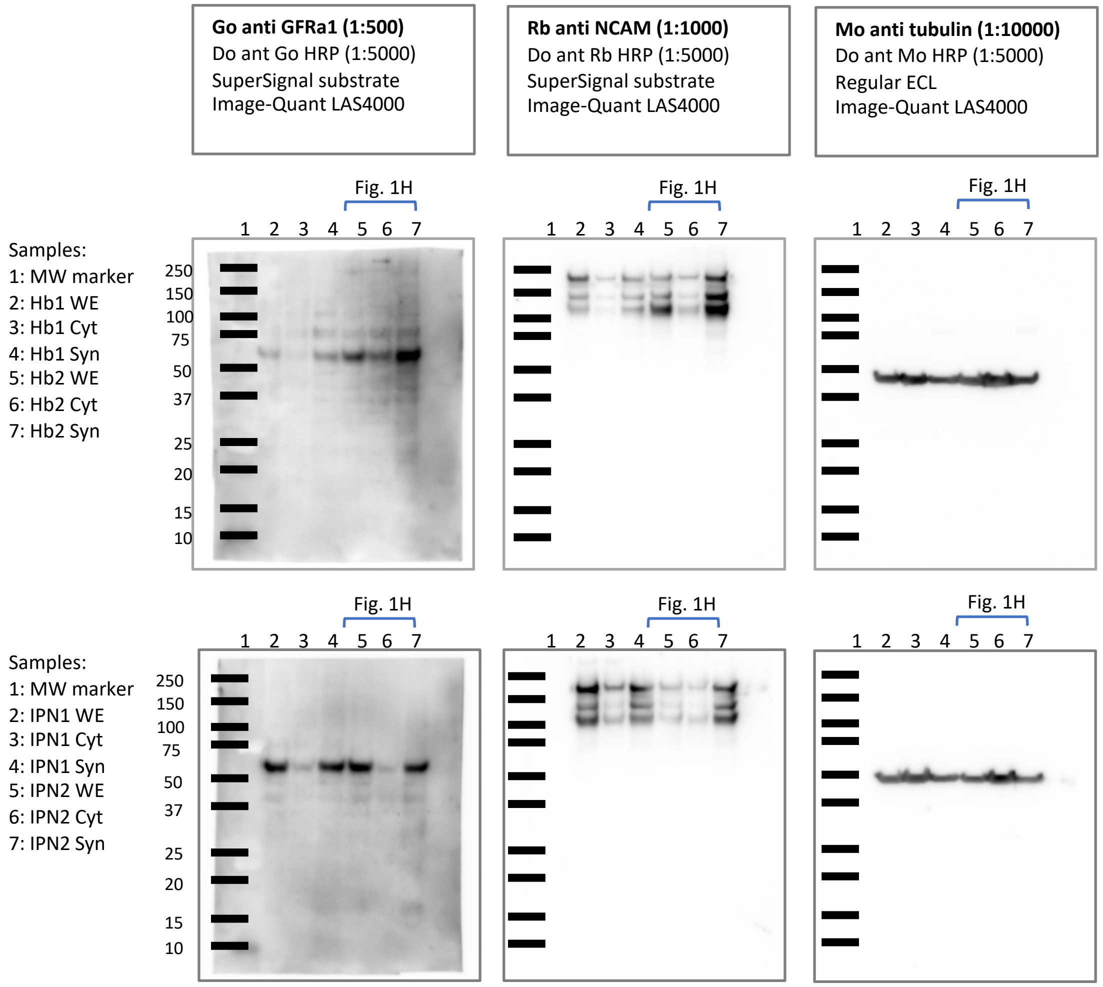

Figure 1I

Samples: mHb and IPN dissected from C57BL6/J mice. The mHb and the IPN of 2 animals were pulled together per sample. The whole extract (WE), the cytosolic (Cyt) and the synaptosome (Syn) fraction were extracted and 20 micrograms of samples loaded in each lane.

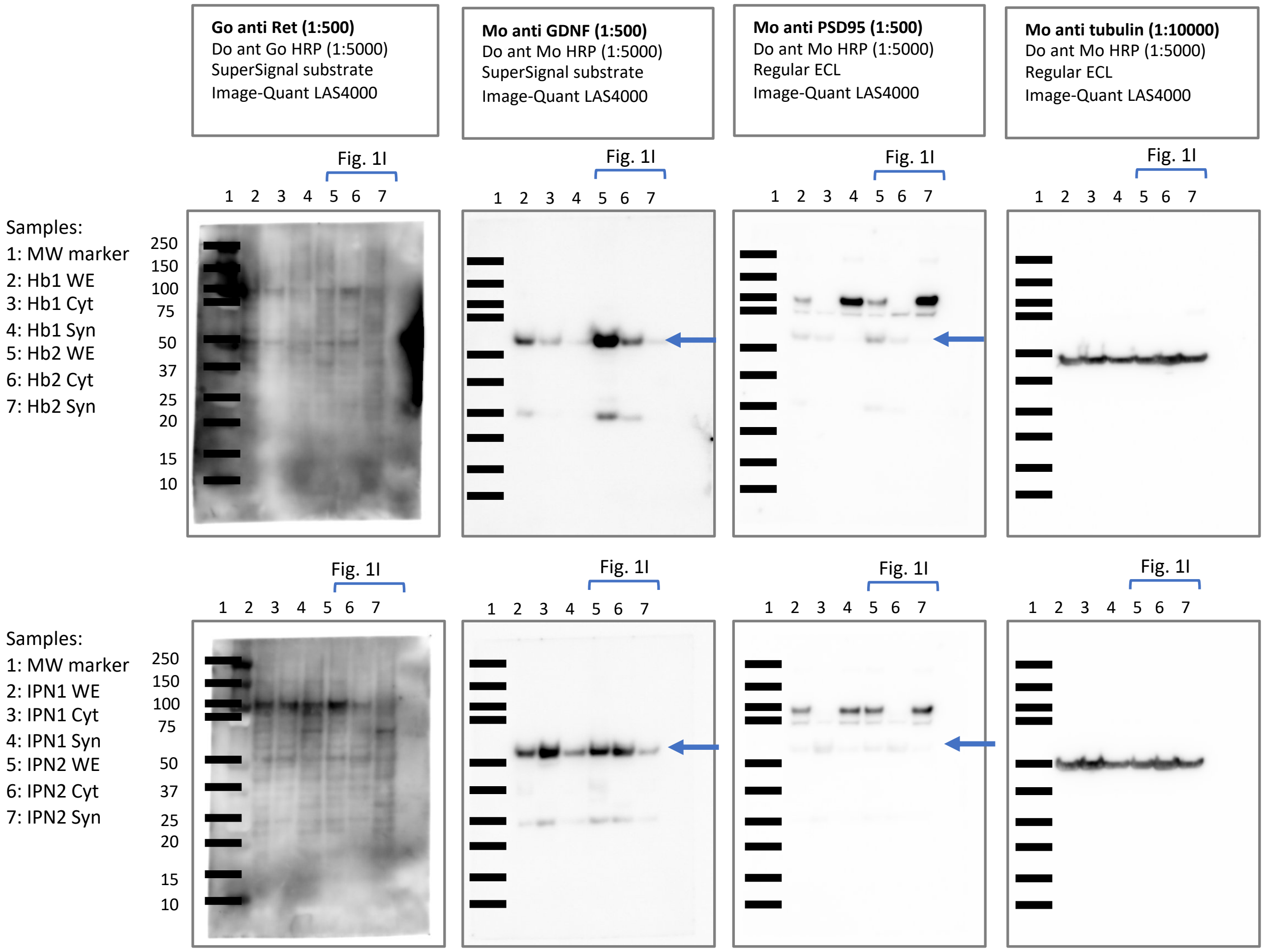

* Indicates inespecific bands not included in the image on the manuscript

Figure 5A. mHb GluA1

Samples: mHb dissected from WT, Het and KO mice. The mHb of 2 animals were pulled together per sample. The whole extract (WE), the cytosolic (Cyt) and the synaptosome (Syn) fraction were extracted and 20 micrograms of the synaptic fraction were loaded in each lane

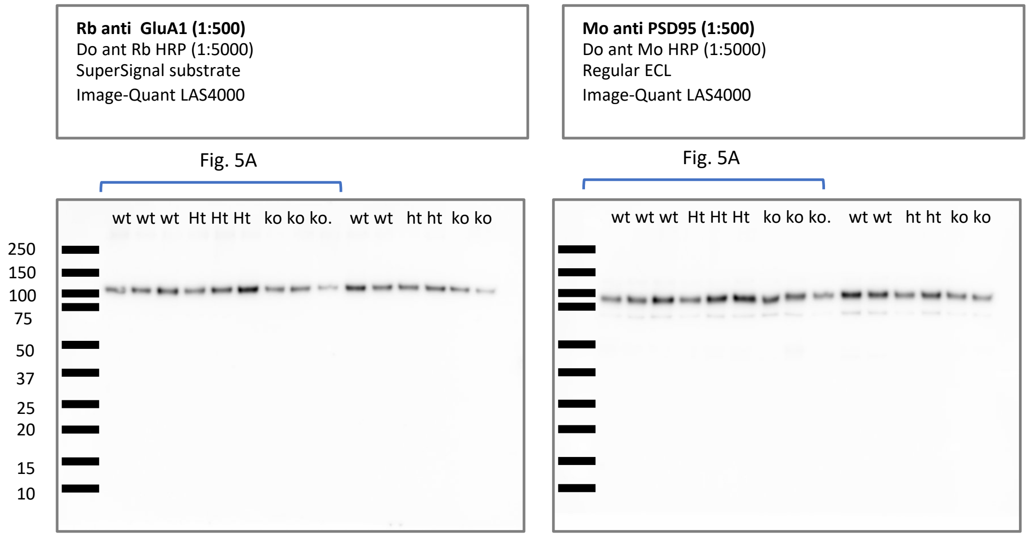

Figure 5A. mHb GluA2

Samples: mHb dissected from WT, Het and KO mice. The mHb of 2 animals were pulled together per sample. The whole extract (WE), the cytosolic (Cyt) and the synaptosome (Syn) fraction were extracted and 20 micrograms of the synaptic fraction were loaded in each lane.

Rb anti GluA2 (1:500)
Do ant Rb HRP (1:5000)
SuperSignal substrate
Image-Quant LAS4000

Mo anti PSD95 (1:500)
Do ant Mo HRP (1:5000)
Regular ECL
Image-Quant LAS4000

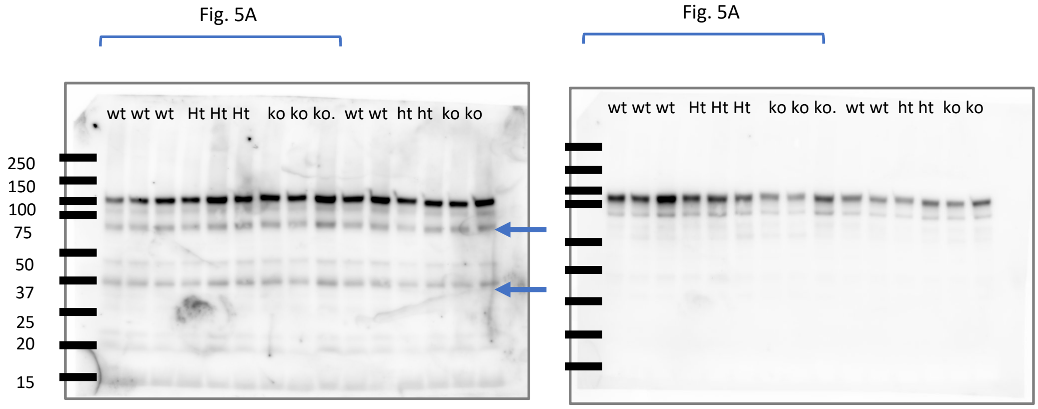

* ← Indicates inespecific bands not included in the image on the manuscript

Figure 5A. mHb GluA3

Samples: mHb dissected from WT, Het and KO mice. The mHb of 2 animals were pulled together per sample. The whole extract (WE), the cytosolic (Cyt) and the synaptosome (Syn) fraction were extracted and 20 micrograms of the synaptic fraction were loaded in each lane.

Rb anti GluA3 (1:500)
Do ant Rb HRP (1:5000)
SuperSignal substrate
Image-Quant LAS4000

Mo anti PSD95 (1:500)
Do ant Mo HRP (1:5000)
Regular ECL
Image-Quant LAS4000

Fig. 5A

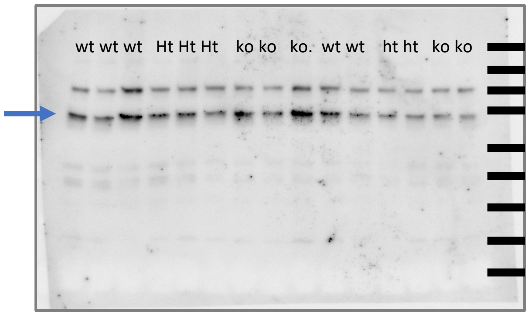

Fig. 5A

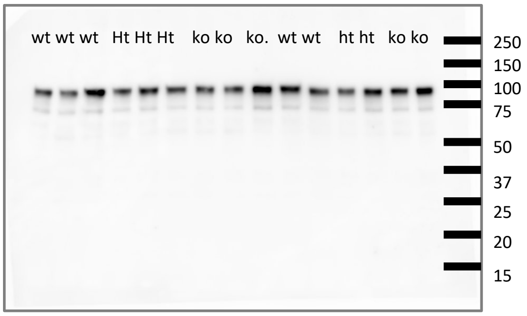

* ← Indicates inespecific bands not included in the image on the manuscript

Figure 5A. mHb GluA4

Samples: mHb dissected from WT, Het and KO mice. The mHb of 2 animals were pulled together per sample. The whole extract (WE), the cytosolic (Cyt) and the synaptosome (Syn) fraction were extracted and 20 micrograms of the synaptic fraction were loaded in each lane.

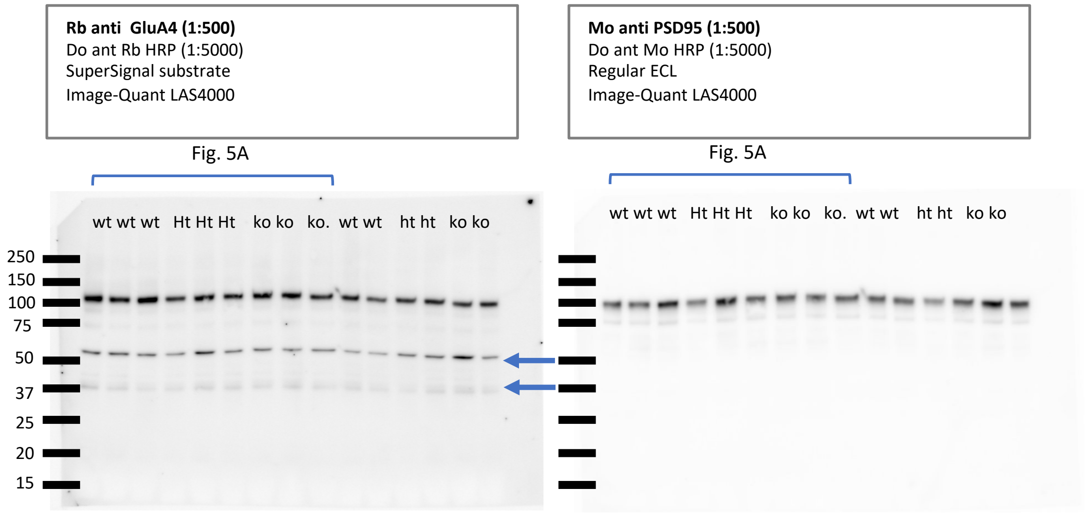

Figure 5B. IPN GluA1

Samples: IPN dissected from WT, Het and KO mice. The IPN of 2 animals were pulled together per sample. The whole extract (WE), the cytosolic (Cyt) and the synaptosome (Syn) fraction were extracted and 20 micrograms of the synaptic fraction were loaded in each lane.

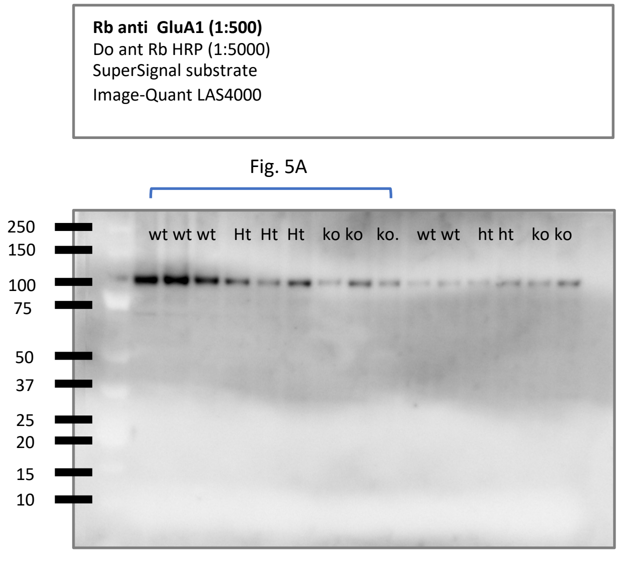

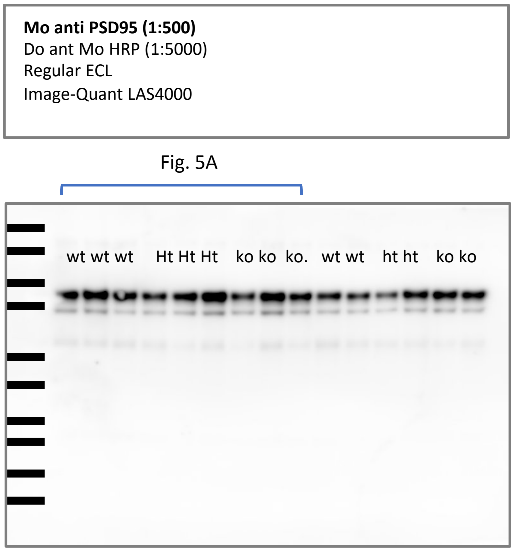

Figure 5B. IPN GluA2

Samples: IPN dissected from WT, Het and KO mice. The IPN of 2 animals were pulled together per sample. The whole extract (WE), the cytosolic (Cyt) and the synaptosome (Syn) fraction were extracted and 20 micrograms of the synaptic fraction were loaded in each lane.

Rb anti GluA2 (1:500)
Do ant Rb HRP (1:5000)
SuperSignal substrate
Image-Quant LAS4000

Fig. 5B

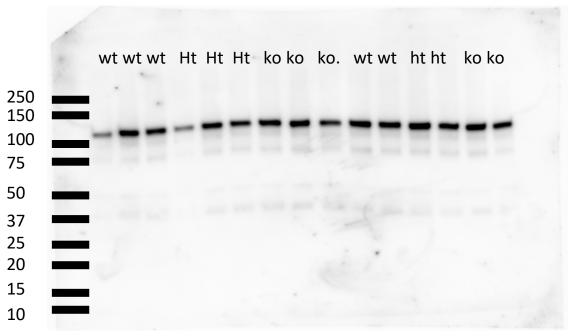

Mo anti PSD95 (1:500)
Do ant Mo HRP (1:5000)
Regular ECL
Image-Quant LAS4000

Fig. 5B

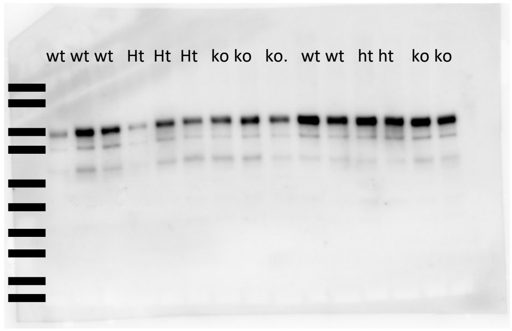

Figure 5B. IPN GluA3

Samples: IPN dissected from WT, Het and KO mice. The IPN of 2 animals were pulled together per sample. The whole extract (WE), the cytosolic (Cyt) and the synaptosome (Syn) fraction were extracted and 20 micrograms of the synaptic fraction were loaded in each lane.

Rb anti GluA3 (1:500)
Do ant Rb HRP (1:5000)
SuperSignal substrate
Image-Quant LAS4000

Fig. 5B

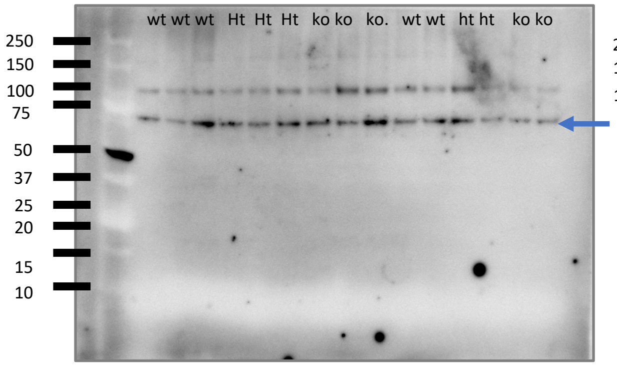

Mo anti PSD95 (1:500)
Do ant Mo HRP (1:5000)
Regular ECL
Image-Quant LAS4000

Fig. 5B

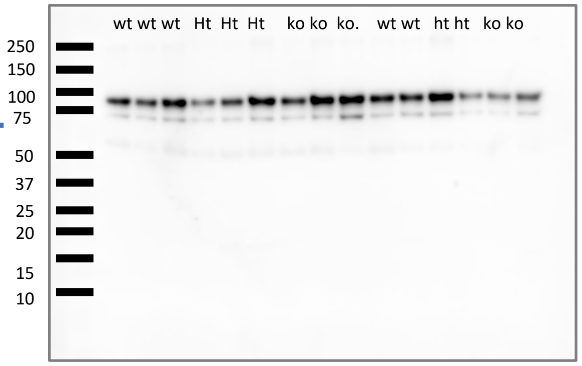

* ← Indicates inespecific bands not included in the image on the manuscript

Figure 5B. IPN GluA4

Samples: IPN dissected from WT, Het and KO mice. The IPN of 2 animals were pulled together per sample. The whole extract (WE), the cytosolic (Cyt) and the synaptosome (Syn) fraction were extracted and 20 micrograms of the synaptic fraction were loaded in each lane.

Rb anti GluA4 (1:500)
Do ant Rb HRP (1:5000)
SuperSignal substrate
Image-Quant LAS4000

Mo anti PSD95 (1:500)
Do ant Mo HRP (1:5000)
Regular ECL
Image-Quant LAS4000

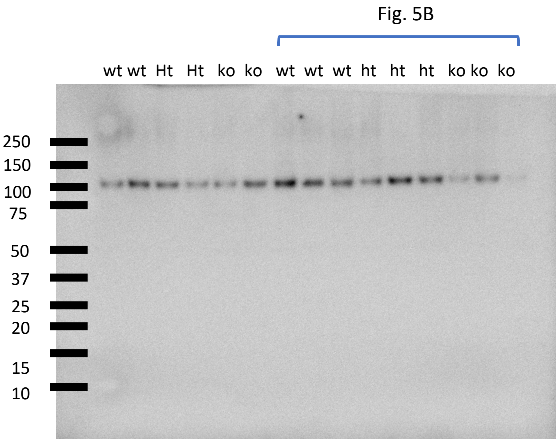

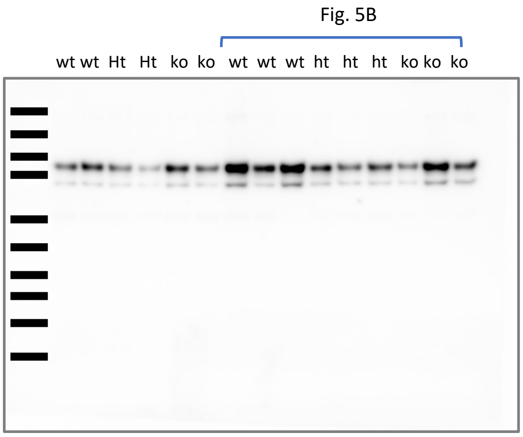

Figure 5C. mHb P-Ser GluA1

Samples: mHb dissected from WT and KO mice. The mHb of 2 animals were pulled together per sample. The whole extract (WE), the cytosolic (Cyt) and the synaptosome (Syn) fraction were extracted and 20 micrograms of the synaptic fraction were loaded in each lane.

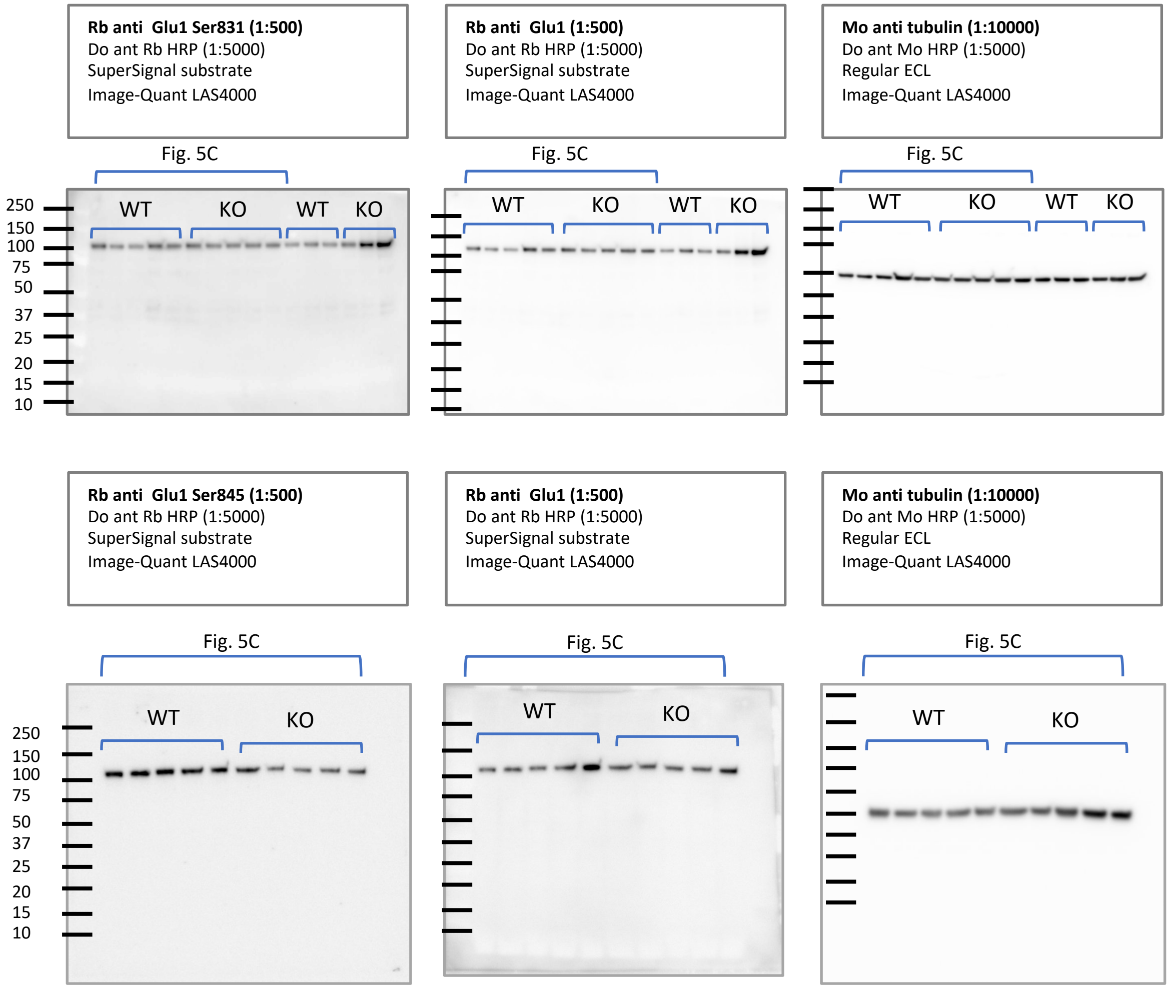

Figure 5E. IPN P-Ser GluA1

Samples: IPN dissected from WT and KO mice. The IPN of 2 animals were pulled together per sample. The whole extract (WE), the cytosolic (Cyt) and the synaptosome (Syn) fraction were extracted and 20 micrograms of the synaptic fraction were loaded in each lane.

Rb anti Glu1 Ser831 (1:500)
Do ant Rb HRP (1:5000)
SuperSignal substrate
Image-Quant LAS4000

Rb anti Glu1 (1:500)
Do ant Rb HRP (1:5000)
SuperSignal substrate
Image-Quant LAS4000

Mo anti tubulin (1:10000)
Do ant Mo HRP (1:5000)
Regular ECL
Image-Quant LAS4000

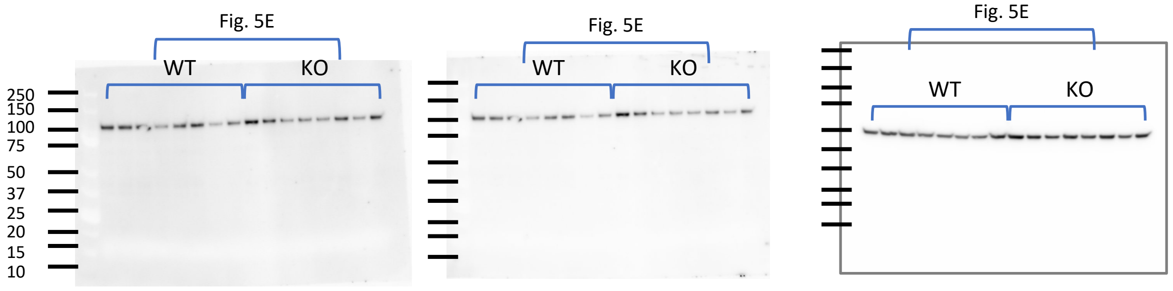

Rb anti Glu1 Ser845 (1:500)
Do ant Rb HRP (1:5000)
SuperSignal substrate
Image-Quant LAS4000

Rb anti Glu1 (1:500)
Do ant Rb HRP (1:5000)
SuperSignal substrate
Image-Quant LAS4000

Mo anti tubulin (1:10000)
Do ant Mo HRP (1:5000)
Regular ECL
Image-Quant LAS4000

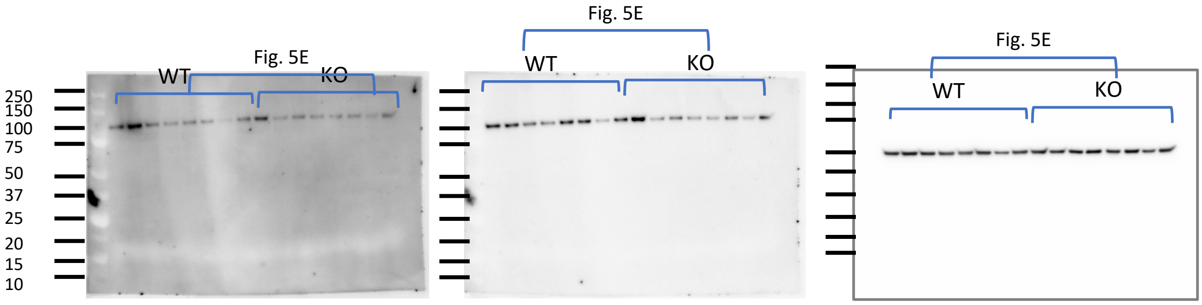

WESTERN-BLOTS SUPPLEMENTAL FIGURES

Figure S1D

Samples: whole protein extract from different brains areas dissected from a C57BL6/J adult mouse.
20 micrograms of sample per lane.

Go anti GFRa1 (1:500)
Do ant Go HRP (1:5000)
SuperSignal substrate
Image-Quant LAS4000

Mo anti actin (1:1000)
Do ant Mo HRP (1:5000)
Regular ECL
Image-Quant LAS4000

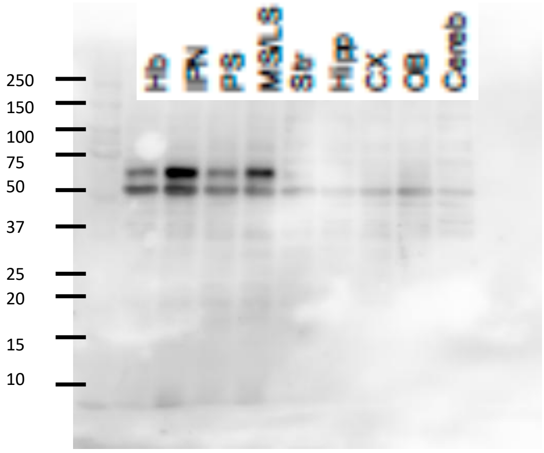

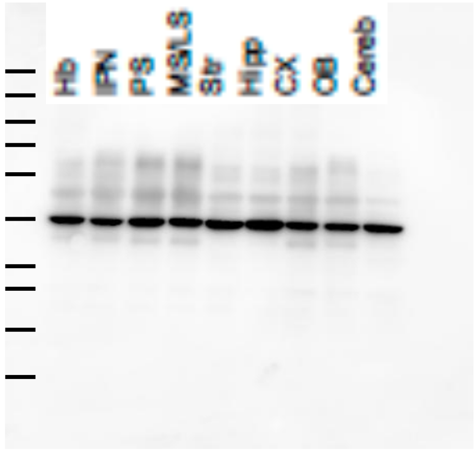

Figure S4F

Samples: whole protein extract from Hb and IPN of WT, Het and KO mice. As a negative control for gfra1, whole protein extract from the Cerebellum of a WT mice was used.
20 micrograms of sample per lane.

Go anti GFRa1 (1:500)
Do ant Go HRP (1:5000)
SuperSignal substrate
Image-Quant LAS4000

Mo anti tubulin (1:10000)
Do ant Mo HRP (1:5000)
Regular ECL
Image-Quant LAS4000

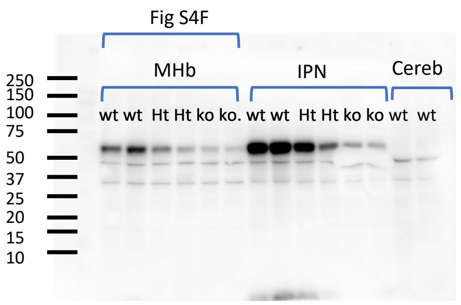

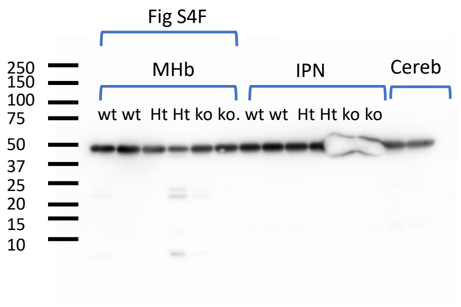

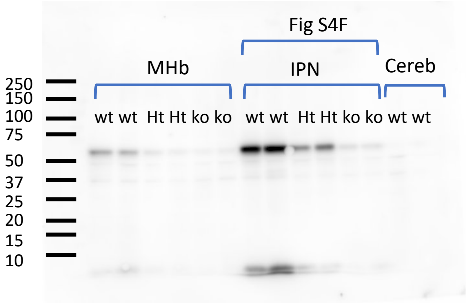

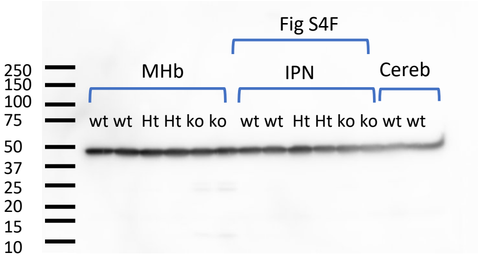

Figure S8B

Samples: Hipp, mHb and IPN from C57BL6/J adult mice were dissected. For the IPN and the mHb, the structure from two mice were pulled together per sample. The whole extract (WE), the cytosolic (Cyt) and the synaptosome (Syn) fraction were extracted and 20 micrograms of each sample were loaded per lane.

Samples (all gels the same):

Hipp	mHb	IPN
WE Ct Syn	WE Cyt Syn	WE Cyt Syn

On Figure S8B only the bands corresponding to the mHb and the IPN are shown

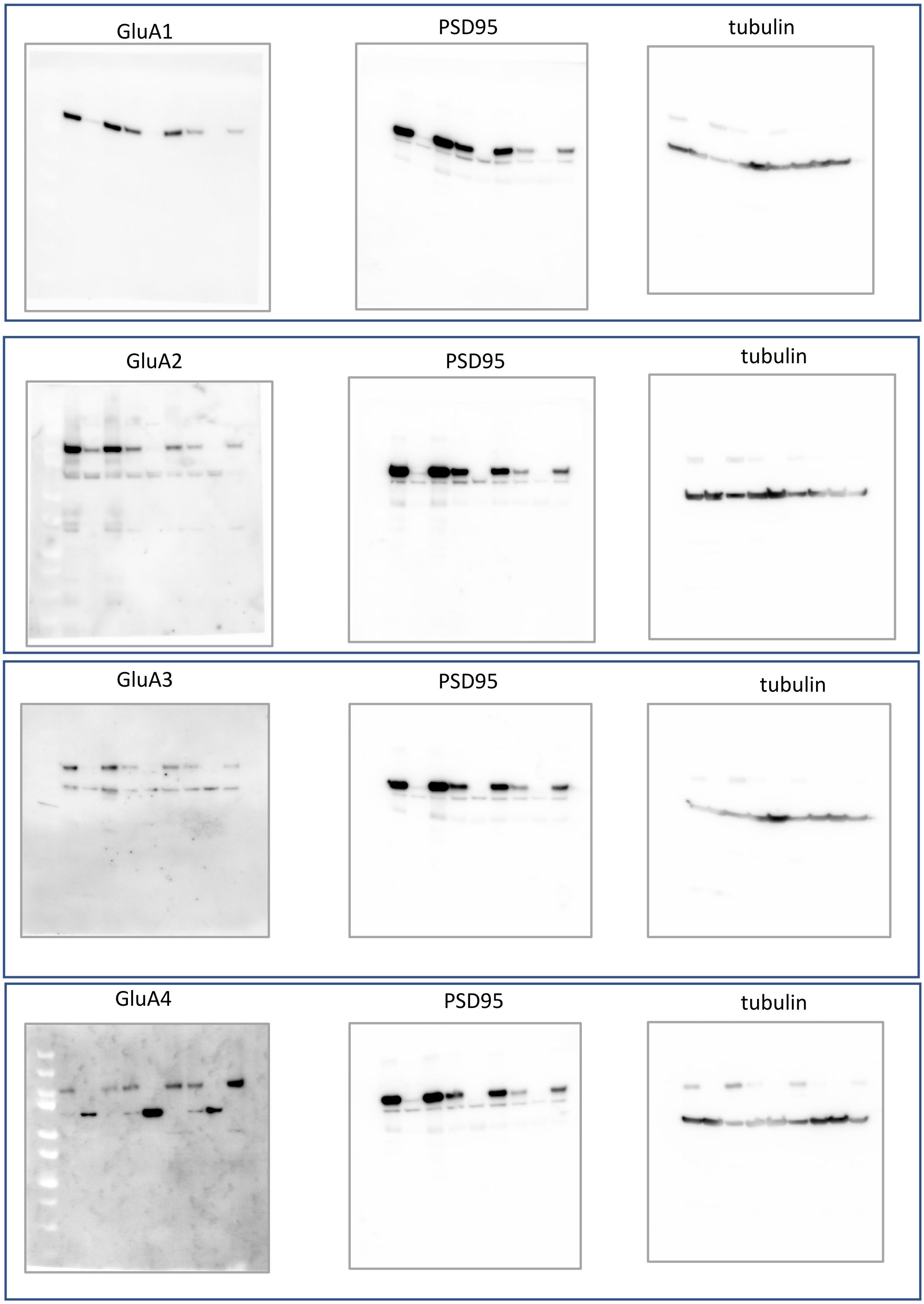

Supplement: S1 Raw Images — (PDF) [file pbio.3001350.s014.pdf]
