# Supplementary material for: Adult medial habenula neurons require GDNF receptor GFRα1 for synaptic stability and function
Source: PLoS Biol. 2021 Nov 8;19(11):e3001350. doi: 10.1371/journal.pbio.3001350 (PMC8601618; doi:10.1371/journal.pbio.3001350)
Supplement: Attachment — Submitted filename: Response to reviewers.pdf [file pbio.3001350.s015.pdf]

RESPONSE TO REVIEWER COMMENTS

Reviewer #1:

This manuscript entitled "Adult medial habenula neurons----------------------------for synaptic stability and function" by Fernandez-Suarez et al., analyzes the role of GFRa1 in the septal-mHb-IPN pathway. This study demonstrates a robust expression of GFRa1 in the mHb. Authors have used a wide range of genetic, chemogenetic, optogenetic, imaging, electrophysiological and behavioral tools to undertake this study. While this study has addressed the effect of GDNF signaling on some of the important synaptic and behavioral aspects of the septal-mHb-IPN pathway, the incorrect/less rigorous interpretation of some of the results diminish enthusiasm for the current version of this manuscript. Also, the discussion section is unfocused and does not adequately convey the importance of the current findings.

Major comments

1. Lack of GFRa1 in the IPN neurons: The expression of tdTomato in the IPN of Gfra1tdTom mice might be due to the mHb axons as the authors claim (as suggested by the mRNA results). Authors use GAD67 cre mice to confirm that only a few IPN neurons show Gfra1. However, it is unclear how much of the GAD67cre neurons represent IPN neurons. Multiple subtypes of GABAergic neurons might be present in the IPN. Therefore, a confirmation is needed that the majority if not all of the IPN GABAergic neurons are labeled in GAD67 cre mice.

It has been previously shown that more than 70% of the GABAergic neurons of the IPN express GAD1 and GAD2 (PMID: 24239118). Furthermore, while GAD67 produces the basal pool of GABA, GAD65 is specialized to respond to short-term increases in demand in synaptic terminals (PMID: 9871412). Therefore, the majority of the GABAergic neurons of the IPN express GAD67. To further evaluate the pattern of expression of *Gfra1* observed using the GAD67Cre line, we have performed RNAscope in brain sections from C57BL6/J adult mice and created a new supplementary figure (Fig S2), showing high levels of *gfra1* expression in the dorsal and ventral mHb, very low levels in the dorsal IPN and no signal in the ventral IPN. This result is in agreement with the observations made with the GAD67Cre line and with the mRNA expression studies by qPCR (Fig. S1B), that clearly indicate minimal or no Gfra1 expression in the IPN.

A relevant issue here is the co-localization of PSD93 and Gfra1, yet the authors claim Gfra1 is pre-synaptic in the IPN.

The level of resolution attained by a confocal microscope at 63X magnification does not allow to distinguish between pre- and post-synaptic sites. Indeed, most studies use co-localization of pre- and post-synaptic markers as empirical evidence of synapses, while it is obvious that the two sides never actually overlap in real life. Thus, in our case, co-localization of GFRa1 with PSD93 only shows that it is located at the synapse, it does not imply a postsynaptic localization. Since GFRa1 is abundantly expressed by mHb neurons, but absent in IPN neurons, our argument for a presynaptic localization seems reasonable. We have postulated that GFRa1 is presynaptic based on its abundant expression in mHb neurons and its absence in IPN neurons. We also note that, as it is common with GPI-anchored proteins, GFRa1 could be released from the presynaptic membrane and bind to co-receptors in the postsynaptic site, leading to non-cell autonomous effects.

Fig 1D shows GFP in areas of the IPN where there is no ChAT or SP staining.

We note that in those images there is a very small percentage of GFP positive areas in the IPN with no ChAT or SP signal. The fluorescent signal for ChAT and SP is really high in some areas of the IPN, therefore it is possible that those fibers are actually positive for those markers but with the set up used to take the images in the confocal microscope the intensity for ChAT and SP is low and they look negative. On the other hand, the IPN receives afferents not only from MHb, but also to a lesser extent from the other brain regions such as median raphe and dorsal tegmental regions (Groenewegen et al., 1986). Therefore, that GFP signal could correspond to fibers from other gfra1 expressing areas that also project to the IPN.

It must be noted that the mHb axonal pattern in the IPN (Ren et al., Neuron, 69: 445-452, 2011; Frahm et al., eLife, 4: e11396, 2015) is very distinct from the distribution of GFRa1 in the IPN.

We have carefully assessed those references and we find that the mHb axonal pattern in the IPN looks very much like the one we observed using ChAT staining in the IPN. We are confident that GFRa1 is present in the somas of the mHb neurons, along the axons and in the axon terminals in the IPN because: i) IHC for GFRa1 shows that the receptor is clearly located in the somas, along the axons of the FR and in the mHb axon terminals in the IPN (Fig. 1E); ii) using the *gfra1*^{Tom} line, we can see strong dTOM signal in the somas, along the axons and in the terminals in the IPN (Fig 1A and S1A,F,G); and iii) using the *gfra1*^{GFP} line, we observe high levels of colocalization of GFP with ChAT and SP in the mHb and the IPN (Fig. 1B,C,D).

The expression of GFRa1 in the lateral part of the IPN is much more intense. These issues need to be addressed when interpreting these results.

While dTOM intensity from the *gfra1*^{Tom} line does appear higher in the lateral IPN than in the core area, immunostaining for endogenously expressed GFRa1 shows a fairly even expression across the IPN (Figs 1E, S3C and S3J). It should also be taken into account that the axonal fibers from the mHb are more densely packed in the lateral IPN than in the core region. This could also contribute to the apparent higher intensity of dTOM signal in that area.

2. Since the authors mention S and crest synapses in the IPN, it is important that figures are labeled appropriately for the readers to see them in these examples. Also, the criteria for the identification of S and crest synapses should be explained.

For quantification of the number of synapses, both S and Crest synapses were included. The S synapses are formed between horizontal axons and small dendrites at asymmetrical contact zones located in the core IPN. The crest synapses are found exclusively in the intermediolateral IPN and are formed by two mHb axons that synapse on opposite sides of dendritic processes between 75 and 80 nm in width (PMID: 6195316). Since the crest synapses are specifically located in a small area of the IPN, the number of crest synapses analyzed was a small percentage of the total synapses. The images on Figure 3C only show S synapses and the graphs shown in Fig 3D correspond to the properties of the S synapses. We have clarified this in the Methods and Results sections, as well as in the figure legend.

3. Expression of dreadd and ChR2: The logic of injecting different viruses expressing cre and DIO-dreadd in the septum is unclear to me. What is the intended target of the virus

expressing cre? A retroviral approach will allow for selectively targeting septal neurons that project to the mHb. Given that the majority of mHb neurons express Gfra1, what is the need for using a cre-based approach in the mHb as it excludes the use of WT group (cre-negative).

We used DREADD constructs that are Cre-dependent, as this allowed for them to be specifically activated in GFRa1-expressing neurons when injected in the mHb in the *gfra1*^{CRE} line. Given the small size and intimate proximity of mHb and LHb neurons, our concern with using Cre-independent constructs was the possibility that DREADD become expressed in LHb neurons (even also in overlying hippocampal neurons), which would have interfered with the results. On the other hand, as septal neurons do not express GFRa1, Cre needed to be supplied externally to those neurons to express DREADD or ChR2. Although a retroviral approach would have allowed us for selectively targeting septal neurons projecting to the mHb, we decided to use the same approach as in previous studies (Otsu et al 2018).

More details are needed for how this study controls for the virus infection/expression of dreadd as these activity experiments involve challenging quantitative comparisons. Also, authors need to present CNO vs vehicle groups comparisons. Given the potential direct effect of CNO or its metabolites (Mahler and Aston-Jones, Neuropsychopharmacology, 43: 934-936, 2018), authors should consider the inclusion of dreadd-free CNO control experiments.

We have added the data of new controls for the DREADD experiments. All these results indicate (i) similar levels of viral expression between groups, (ii) no cFOS expression after CNO treatment in the absence of the DREADD and (iii) no cFOS expression in the presence of the DREADD and the absence of CNO.

Supp. Fig S6 shows the controls of the TS-mHb experiment:
(i) Quantification of the number of GFP+ and cFOS+ cells in the TS of WT, Het and KO mice after CNO treatment.
(ii) OD for mCherry in the terminals in the mHb.
(iii) cFOS counts in the TS and the mHb after CNO treatment in the absence of the DREADD.

Supp. Fig S7 shows the controls for the mHb-IPN experiment:
(i) Quantification of the number of cFOS+ cells after CNO treatment and the OD for mCherry in the mHb of WT, Het and KO mice injected with the DREADD.
(ii) cFOS expression in the mHb and the IPN after CNO treatment in the absence of the DREADD.
(iii) Expression of the DREADD in the absence of tamoxifen.
(iv) cFOS expression in the mHb and the IPN in the presence of the DREADD and the absence of CNO.

4. The changes in calcium permeable AMPA receptors: Based on the data shown, the change in rectification index in this study is due to the modification of AMPA current at -60mV and not due to changes in AMPA current at depolarized conditions as all these groups appear to show significant inward rectification. Assuming that there is no noticeable inconsistency in reverse potential among groups/neurons and therefore no errors in the calculation, it is possible that both the increased amplitude at -60mV and an increased rectification index are due to high synaptic calcium permeable AMPA receptors in WT compared to HET and KO groups. A comparison of mEPSC amplitude might reveal whether there is an increase in AMPA receptor conductance while calculation of rectification index in

cells (from these three groups) with similar EPSC amplitudes (-60mV) obtained after adjusting light intensity/duration will confirm these results.

Unfortunately, in the mHb a non-stationary fluctuation analysis of AMPAR-mediated synaptic transmission is not possible for 2 reasons. First, as we showed in a previous publication (Otsu et al, 2018, Cell Reports, PMID: 29346), mHb cells receive inputs from septal glutamatergic axons so that the responses are basically all-or-none. This implies that modulating their amplitude by adjusting the power of the stimulating light is not possible. Second, and probably not unrelated to the previous argument, an analysis of spontaneous EPSCs (sEPSCs) is not possible in mHb neurons because sEPSCs are totally absent in these cells. After hundreds of whole cell recordings of mHb cells performed in recent years, we never observed spontaneous glutamatergic activity.

Biochemical studies show changes in AMPA receptor subunits in response to manipulation of Gfra1. However, the results are descriptive in nature and therefore, authors should avoid strong claims regarding the calcium permeability of AMPA receptors. Current results are far from establishing any mechanistic understanding of the role of GFRa1 in AMPA receptor regulation or septal-mHb-IPN function.

We respectfully disagree with this statement and reject any pejorative use of the descriptive/mechanistic dialectic. Everyone in the scientific community ought to make an effort to refrain from making such sweeping judgements which are both void of substance and unhelpful to the progress of science. What is descriptive and what is mechanistic? (And, by the way, what's wrong with descriptive results?) The changes in composition and interaction in AMPAR subunits that we observed are very much mechanistic, as they provide a molecular explanation for the electrophysiological changes observed in these synapses. It is true that we do not currently know how GFRa1 affects AMPAR subunits, although we suspect it is by direct interaction. Now, would that be descriptive or mechanistic? Where does this end? As Prof. Eve Marder, co-founder of *eLife*, wrote in a recent editorial, "*When a reviewer asks for mechanism in a paper, I worry that it really means 'You have to do more just because you haven't done enough'.*"

5. Behavior: The foot-shock methods used in the current study and the interpretation of the results from these behavioral experiments are not entirely consistent with the current knowledge in the field. The lack of details in the method section and brief legends do not provide enough information to understand the behavioral procedures. The presented animal studies can only measure threat responses and not innate fear. Also, the descriptions in the manuscript are not clear about threat learning and memory. Panels in figures 6 and S7 are not clear about learning and memory. Authors also do not discuss the inconsistency in the data (Figures 6H and S7D).

The foot-shock assays we have used are also common in other studies that investigated behavioral responses regulated by the septum→mHb→IPN circuit. We have added more details in both the Methods sections and the Figure Legends.

With regards to the discussion of terminology juxtaposing threat vs. fear and innate vs. learnt responses, we note that this is indeed a minefield, and the literature is plagued with controversy and debate, mostly semantic, about the use of such terms. As we see it, fear is a response to an anticipated threat; some scholars may choose to call this a "threat response", and that is fine. At the same time, fear responses can be innate or learnt. We have considered the freezing caused by three consecutive foot-shocks as an innate fear response, since the animal had not previously been exposed to that threat. On the other hand, in the passive avoidance, context and cued-fear conditioning assays, we considered

freezing responses to a specific context or auditory cue (which the animal had previously learnt to associate with a foot-shock) as learnt fear, since in this case the animal was not actually exposed to the threat, but to the conditioning stimulus.

Finally, there is no inconsistency between Figures 6H and S7D. Figure 6H shows freezing responses 24 hours after the training session and in the absence of foot-shock (what we would call learnt fear). On the other hand, panel S7D shows the freezing responses to 3 consecutive foot-shocks (i.e. innate fear), in which we observed no differences between genotypes. This result is in agreement with our observations during the training period of the context fear conditioning assay (shown in Fig. S9H in the revised version of the m.s.). Here, mice received one foot-shock and no differences were observed in freezing responses following the shock.

6. NMDA EPSC: NMDA EPSC is measured at 7 ms post-peak. Such an early decay of AMPA current is surprising (Adesnik et al., PNAS, 105 (14): 5597-602, 2008). Authors should show the data that AMPA currents are completely decayed at 7 ms. Alternatively, a comparison can be made at a later time point.

The decay time constant of the AMPAR-mediated components recorded at +50mV (as measured after pharmacologically blocking the NMDAR-mediated part) amounts to 1.5±0.2ms (N=16; mean ± SEM). Our measure at 7ms following the synaptic response peak thus corresponds to around 4.7 times this value, at which less than 1% of the original amplitude is still present. We are thus very confident of measuring the NMDAR-mediated EPSC properly.

7. Statistical analysis: The information about the number of mice (number of females and males)/slice/samples/cells in each group, the exact P and F values should be available to the reader.

We have now added the N and exact P and F values as a Table in the Supplementary Information.

8. Discussion: A rigorous evaluation of the current results might show some important findings regarding the role of GRFa1 in the septal-mHb-IPN pathway. However, such an effort is lacking in the discussion. The discussion gives the impression that GFRa1 is important for fear memory regulation. Data show that an alteration of the septal-mHb-IPN pathway as a result of robust manipulation of GFRa1 affects the behavior. This does not mean a direct role of GFRa1 in these behaviors. Authors do not make an effort to discuss the basis of decreased anxiety and an increased threat response behavior. While it is acceptable to discuss the earlier studies showing similar behavior effects in response to specific manipulation of septal inputs, the current study using non-specific molecular manipulation of neurons is not comparable to those studies. Apart from the descriptive biochemical results, there are no clear results that mechanistically link GFRa1 to calcium permeability of AMPA receptors. Authors should also consider the fact that these data come from a possible heterogenous groups of neurons. Nevertheless, these results show that a diminished GFRa1 activity affects the AMPA receptor subunits. Therefore, authors should focus on the current results and avoid discussion about a direct role of GFRa1 in favoring the formation of calcium permeable AMPA receptors.

First of all, we would like to note that, in our opinion, selection of topics for Discussion is first and foremost a privilege of the authors. A Discussion section ought to put aspects of

the work in the perspective of previous results, as well as present implications of the results as informed speculations. The authors have the right and duty to choose what aspects of their work they deem more valuable or interesting for such discussion. Informed speculations are valuable motors of scientific progress, even though they remain, well, speculative.

With that out of the way, let us try to clarify our views on a few of the points raised by the reviewer here. First, we are being criticized for speculating about the role of GFRa1 in fear memory regulation. And then for not "*making an effort to discuss the basis of decreased anxiety and an increased threat response behavior*". We find this contradictory, as nothing in our data would seem to justify a balanced and informed speculation about the basis of such dichotomy. As for the point of whether GFRa1 has "direct" effects on behavior, our view is that no single protein can have a direct effect on any behavior. All protein molecules, including GFRa1, can only affect behavior indirectly, through regulation of the structural and/or functional properties of neuronal connections. The reviewer acknowledges that the behavioral effects we observed phenocopy lesions of the septum-mHb pathway described in previous studies. In our view, the fact that adult deletion of one gene, i.e. *Gfra1*, mimics the effects of those lesions is quite striking. In relation to this comparison, however, the reviewer pejoratively refers to our studies as a "*non-specific molecular manipulation of neurons*". This is quite shocking. We have used a series of highly specific knock-in mouse lines expressing CRE, GFP or dTOM under the specific control of our gene of interest, namely *Gfra1*. We have used highly specific stereotaxic injection of CRE-dependent viruses into the region of interest, namely mHb, which can only be activated by CRE that is specifically expressed in those neurons. What if anything is "*non-specific*" in those manipulations please? Regarding the possible heterogeneity of mHb neurons, our view is that most, if not all neurons in the mammalian brain are indeed heterogenous; not two neurons are completely identical in their molecular and structural properties. On the other hand, the mHb neurons we have studied all share one property, namely they all express GFRa1. Our study is about that one property that all these neurons have in common. Finally, given its unusually high abundance (unusual for a signaling protein), we speculated in our paper that GFRa1 may have structural roles at the synapse. We find this intriguing, thought-provoking, and a reasonable and justified point for speculation in the Discussion.

9. References: Many statements throughout the manuscript lack references.

We have now added more references to the revised version of the manuscript.

Minor comments

1. The study of PSD95 vs PSD93 needs to be clearly justified in the manuscript.

It is a well-known fact in the field that, while commercial PSD95 antibodies work quite well for Western-blotting, they are not nearly as good for histological analyses. As in many other similar studies, we chose to use PSD93 as a glutamatergic synaptic marker for the quantification of synaptic puncta, as antibodies to this target work quite well for immunohistochemistry.

2. Fig S3b does not show DAPI.

We included DAPI only in the images corresponding to the animal injected with the vehicle of tamoxifen. In the pictures corresponding to the mouse injected with tamoxifen both the mHb and the IPN are clearly recognizable by looking at the dTOM signal and therefore we did not include DAPI. We have modified the figure legend to clarify this point.

3. Lateral IPN vs core IPN, details of core IPN should be included.

It is not clear what the reviewer means with this. Higher magnification images? Text descriptions?

Reviewer #2, Alberto Pascual:

The paper by Fernández-Suárez et al. identifies the role of GFRa1 in synaptic stability and function. In general, the paper is very interesting, combining mouse models with viral injections that allow the authors to define the role of this protein in the septum-habenula-IPN circuit. The data are of quality and the experiments are well described. My opinion is very positive with several minor points:

1. Tamoxifen treatment may alter the behavior of mice. I only found in the M&M sections that all the groups were treated with TMX when describing the electron microscopy procedure. Please, specify in the mice section that all the groups (WT, Het, and KO mice) were treated with TMX. If that was not the case, at least behavioral experiments should be repeated with TMX treatment to all the experimental groups.

All animals received TMX. We have now clarified this point in the Methods section.

2. Quantification of electron microcopy images is not described in the M&M section, please include this information, including the number of synapsis analyze per mouse (also to be included in the figure legend). Regarding presynaptic vesicles/per vesicle pool, please define what is the meaning of "vesicle pool" (regarding the quantification). Was the quantification normalized to the area of the presynaptic boutons analyzed?

The number of vesicles was not normalized to the area of the synaptic boutons, but we did not observe differences in overall synapse size, as indicated by similar PSD length among genotypes. We have now included a more comprehensive description of the quantification of the electron microscopy images in the Methods section and the number of synapses analyzed per mouse in the figure legend.

3. Regarding the analysis of the functional connectivity, line 258 should include the possibility of a role of GFRa1 during development (circuit establishment), as no differences were found between Het and KO mice (Fig. 4C).

This point, as well as the three following points, all deal with some of the observed differences between WT and HET mice and whether such differences suggest possible haplo-insufficiency of GFRa1 during development. Indeed, in several of our studies with the *Gfra1*^{Cre} line, HET mice showed and intermediate effect between WT and KO. We have now clarified this in the revised text of the manuscript.

4. A trend is observed in SF5B and F regarding c-Fos expression, please add the p-value in those experiments (WT vs Het). This could suggest a developmental role of GFRa1 in the establishment of the circuit (in addition to the well-described phenotype already shown in other figures).

All p- values have been compiled in a new Table included in the Supplm Information. p-values between WT and Het in the experiments shown in Figs. S5B and F are p=0.0627 and p=0.1892, respectively.

5. Please, also include the p-value in Fig. 4GI between WT and Het groups. To claim an adult role of GFRa1, the Het and the KO should be different, as the Het is the right control of the experiment (same genotype except in adult deletion of the Floxed allele).

We did not observe significant differences between the WT and HET groups in this case (p= 0.3625 in Fig. 4G and p=0.2706 in Fig. 4I). As mentioned, all p- values have been compiled in a new Table included in the Supplm Information.

6. In Figure 5, I find the same problem, no differences are found between the WT and Het groups, but also no differences between Het and KO. In Fig. 5C-J, Het mice are directly excluded. Although that does not compromise the message of the article (Fig. 2E-H and Fig. 6 clearly demonstrate an adult role for GFRa1), the potential role of GFRa1 during development should be further discussed.

See response to point 3 above.

7. Our experience with antibodies against GDNF is not very positive. We have tested many of them using brain extracts from P0 WT and GDNF-/- mice and many of them recognize a band of 25 kDa present in both samples. I would kindly ask the authors to check the specificity of the GDNF antibody used and, if the band is not present in the P0 mutant extract, add the control to a supplementary figure to clarify that this is a perfect antibody. On the contrary, the figures with GDNF in western blot should be removed from the article and either substitute with ELISA or just show the q-RT-PCR data.

We have removed the western blot for GDNF and added the quantification using ELISA (Fig. S1E).

8. Line 120, this affirmation is controversial, see PMIDs: 18536709, 25710829, and 31930748.

Thanks. We have modified that sentence (p.6 line 118) and cite those references in the revised version of the manuscript.

9. Lines 139 and 152, please cite PMID: 27445711.

We added the citation indicated in the revised version of the manuscript.

10. Please, homogenize through the text the way of presenting the p values (p < 0.00), as many different options are already stated (p<0.00; p <0.00; p< 0.00). Same thing with units (X mM instead of XmM).

Thanks for this heads-up. We have done so.

11. In line 508, please change "H2O"
12. In line 609, please change "um" to "µm"

Thanks. Both typos have been corrected in the revised version of the manuscript.

Reviewer #3:
In this work, Fernandez-Suarez et al revealed the function of the GFRalpha1 in the medial habenula circuits using a multidisciplinary approach. Overall, I enjoyed the manuscript and all the data the authors were able to gathered. It is really a great work. I do have some concerns that I am sure the authors will be able to answer.

About the AAVs injections in the MHb, the injection was bilateral but only in one anterior-posterior (AP) coordinate, right? If so, the deletion was observed along the full MHb? or only in one part? Did the AAVs reach other brain regions? is the expression of Gfra1 in the MHb constant along the AP MHb??

We only use one AP coordinate. Deletion was observed along the full mHb, but there remained some areas further away from the injection site that still contained some receptor. As show in Fig S3J the deletion of the receptor was not 100% in the virus model. For this reason, we used mHb sections separated by 120um from bregma -1.06 to -2.18 for the histological experiments, thereby encompassing the whole nucleus. The AAVs also reached limited areas in the LHb and the hippocampus along the injection path. Since GFRa1 is not expressed by LHb neurons and the number of affected hippocampal cells was very low, we consider this injection to be mHb specific. Injections that deviated significantly from this pattern were discarded.

The authors checked the expression of any cholinergic marker in their experiments? If not? why? would you expect the same changes as the glutamatergic ones?

It has been proposed that axon terminals from the mHb neurons in the IPN co-release glutamate and ACh (PMID: 21315256), and therefore it would be possible that in the absence of gfra1 the levels of expression of cholinergic markers could be altered as well. However, we examined the levels of VChAT in the mHb terminals of the dorsal, ventral and lateral subnuclei of the IPN, but detected no differences between WT, HET and KO mice. Those data are now shown in Fig. S5E,F and a description of the result has been added in the revised version of the m.s. (p. 10 line 221).

In Figure 4B (cFos):in WT you have both dorsal and ventral Fos, in HET mostly dorsal, in KO mostly ventral (also maybe in the PVT).
why?? is it juts my impression or is it constant? any double labelling planned? CHAT,Vglut?

We have re-assessed these images and this is not constant. In all cases most of the cFOS positive cells are located in the ventral part of the mHb, which receive the projections from the TS. We have included a different image of the Het in Fig. 4B.

Page 15, line 347: why onlt 2 weeks? would you expect different results after 5 weeks for example?

We evaluated the decay in glutamatergic synaptic markers at 7, 10, 14 and 28 days following tamoxifen treatment. While no differences were seen at 7 days, a trend appeared at 10days. At 14 days, the decay was very similar in magnitude to that observed 28 days after tamoxifen treatment. We therefore decided to begin the behavioral evaluation 2 weeks after tamoxifen treatment, since the synaptic phenotype was already present at that time. We did not include all this analysis in the final version of the paper, as we believed it did not add any relevant information. It is possible that stronger behavioral alterations would be detected at later time points.

Page 15, line 348: nor sure if it is correct to say that you evaluate anxiety ? they are just the innate responses to potential danger. right? I mean, the behavior is not pathological.

We agree that innate responses to a potential danger or threat are not pathological, and therefore a more appropriate term would be “anxiety-like behaviour”. We have made this change in the revised text.

Do you have the distance measurement in the EPM?

Yes, we do. We did not observe significant differences in the distance covered between groups. These data have been added in Fig. S9C,D.

You mentioned that the KO mice show a trend towards increased distance travelled in the OF: it would be crucial to to make an analysis correlating the data obtained in the OF with the EPM (distance for example). To see if the mice "traveling" more are the same "traveling " more in the other tests. Because in some graphs it seems that you have two very distinct groups (e.g., latency in AA). Is it because the expression off the Gfra1 is different in habenular circuits? Do you evaluate the expression the receptor after the behavioral paradigms?

In the open field (OF), we observed a trend towards increased distance travelled in the center of the arena in the mHb.KO mice, while there were no differences in the total distance. We have performed correlation analysis between the total distance travelled in the OF and total distance in the EPM, as well as between the distance in the center of the OF and that in the open arms of the EPM, but could not detect any significant correlation in either case. These data have been added in Fig. S9E,F.

We did evaluate the expression of the receptor after the behavioral analyses, but did not observe differences by immunohistochemistry compared to mice that were not subjected to behavioral analyses. The KO mice showed no expression of *Gfra1* and the HET mice showed about half of the levels of the receptor compared to WT mice.

Did the authors evaluate pain perception?

Preclinical data have implicated the habenula in numerous behaviors including pain and analgesia. Although it would be of interest to evaluate pain perception in our model, we have not performed those experiments.

the authors didnt find differences in CFC, but there are differences in Passive avoidance? which also uses a CUE (am I right), which I think in this case is visual, right? so maybe there is a specifcity about the type of cue? does it make sense?

In this study we performed two tests to measure contextual fear conditioning (passive avoidance and context fear conditioning), and detected differences in the mHb.KO mice. On the other hand, we performed one test to evaluate auditory fear conditioning (cued fear conditioning), in which no differences were observed. Therefore, it would seem that the behavioral deficits observed in KO mice affect specifically the contextual fear conditioning. It has been suggested that the amygdala has an important role in both acquisition and expression of auditory fear conditioning, while the hippocampus is necessary for contextual fear conditioning. As the septofimbrial and triangular septum receive their major inputs from the hippocampal formation, it is therefore possible that lesions or functional alterations in the mHb affect contextual fear conditioning specifically.

Finally, did the authors try to evaluate any APPETITIVE conditioning (or response)? is it specific of AVERSIVENESS?

We have focused the present work on behavioral paradigms that had previously been shown to be affected by lesions in the septo-habenular pathway. We agree that appetitive conditioning would be an interesting direction for future studies.

Again, congratulations for the massive work.

Thank you very much.
