# Supplementary material for: Adult medial habenula neurons require GDNF receptor GFRα1 for synaptic stability and function
Source: PLoS Biol. 2021 Nov 8;19(11):e3001350. doi: 10.1371/journal.pbio.3001350 (PMC8601618; doi:10.1371/journal.pbio.3001350)
Supplement: Attachment — Submitted filename: RESPONSE TO REVIEWER COMMENTS.pdf [file pbio.3001350.s016.pdf]

RESPONSE TO REVIEWER COMMENTS

Reviewer #1: I have no further comments regarding this paper.

Reviewer #2, Alberto Pascual: The authors have addressed all my initial concerns. Two notes: 1) Western blot for GDNF should be removed in Fig1I, as the authors did not validate the antibody and the information is not relevant for the article.

We have removed the western blot for GDNF in Fig1 and the sentence including its description in the Results section.

2) In page 10-line 216, the sentence can be misleading, as no significant differences were found between HET and KO mice, so better remove "compared to Het or WT" and use "compared to WT".

We have change that sentence and use “compared to WT”.

Reviewer #3, Edgar Soria-Gómez: I have no further comments.
